# Feasibility of using low-cost markerless motion capture for assessing functional outcomes after lower extremity musculoskeletal cancer surgery

**DOI:** 10.1371/journal.pone.0300351

**Published:** 2024-03-28

**Authors:** Sherron Furtado, Brook Galna, Alan Godfrey, Lynn Rochester, Craig Gerrand

**Affiliations:** 1 Department of Orthopaedics and Musculoskeletal Science, University College London, London, United Kingdom; 2 Therapies and Department of Orthopaedic Oncology, London Sarcoma Service, Royal National Orthopaedic Hospital NHS Trust, Stanmore, United Kingdom; 3 School of Allied Health (Exercise Science), Murdoch University, Perth, Australia; 4 Translational and Clinical Research Institute, Newcastle University, Newcastle upon Tyne, United Kingdom; 5 Computer and Information Science Department, Northumbria University, Newcastle upon Tyne, United Kingdom; 6 Newcastle upon Tyne Hospitals NHS Foundation Trust, Newcastle upon Tyne, United Kingdom; 7 Department of Orthopaedic Oncology, The London Sarcoma Service, Royal National Orthopaedic Hospital NHS Trust, Stanmore, United Kingdom; University of Verona: Universita degli Studi di Verona, ITALY

## Abstract

**Background:**

Physical limitations are frequent and debilitating after sarcoma treatment. Markerless motion capture (MMC) could measure these limitations. Historically expensive cumbersome systems have posed barriers to clinical translation.

**Research question:**

Can inexpensive MMC [using Microsoft Kinect^TM^] assess functional outcomes after sarcoma surgery, discriminate between tumour sub-groups and agree with existing assessments?

**Methods:**

Walking, unilateral stance and kneeling were measured in a cross-sectional study of patients with lower extremity sarcomas using MMC and standard video. Summary measures of temporal, balance, gait and movement velocity were derived. Feasibility and early indicators of validity of MMC were explored by comparing MMC measures i) between tumour sub-groups; ii) against video and iii) with established sarcoma tools [Toronto Extremity Salvage Score (TESS)), Musculoskeletal Tumour Rating System (MSTS), Quality of life-cancer survivors (QoL-CS)]. Statistical analysis was conducted using SPSS v19. Tumour sub-groups were compared using Mann-Whitney U tests, MMC was compared to existing sarcoma measures using correlations and with video using Intraclass correlation coefficient agreement.

**Results:**

Thirty-four adults of mean age 43 (minimum value—maximum value 19–89) years with musculoskeletal tumours in the femur (19), pelvis/hip (3), tibia (9), or ankle/foot (3) participated; 27 had limb sparing surgery and 7 amputation. MMC was well-tolerated and feasible to deliver. MMC discriminated between surgery groups for balance (p<0.05*), agreed with video for kneeling times [ICC = 0.742; p = 0.001*] and showed moderate relationships between MSTS and gait (p = 0.022*, r = -0.416); TESS and temporal outcomes (p = 0.016* and r = -0.0557*), movement velocity (p = 0.021*, r = -0.541); QoL-CS and balance (p = 0.027*, r = 0.441) [* = statistical significance]. As MMC uncovered important relationships between outcomes, it gave an insight into how functional impairments, balance, gait, disabilities and quality of life (QoL) are associated with each other. This gives an insight into mechanisms of poor outcomes, producing clinically useful data i.e. data which can inform clinical practice and guide the delivery of targeted rehabilitation. For example, patients presenting with poor balance in various activities can be prescribed with balance rehabilitation and those with difficulty in movements or activity transitions can be managed with exercises and training to improve the quality and efficiency of the movement.

**Significance:**

In this first study world-wide, investigating the use of MMC after sarcoma surgery, MMC was found to be acceptable and feasible to assess functional outcomes in this cancer population. MMC demonstrated early indicators of validity and also provided new knowledge that functional impairments are related to balance during unilateral stance and kneeling, gait and movement velocity during kneeling and these outcomes in turn are related to disabilities and QoL. This highlighted important relationships between different functional outcomes and QoL, providing valuable information for delivering personalised rehabilitation. After completing future validation work in a larger study, this approach can offer promise in clinical settings. Low-cost MMC shows promise in assessing patient’s impairments in the hospitals or their homes and guiding clinical management and targeted rehabilitation based on novel MMC outcomes affected, therefore providing an opportunity for delivering personalised exercise programmes and physiotherapy care delivery for this rare cancer.

## 1. Introduction

Multi-modal management for lower extremity musculoskeletal tumours (bone and soft tissue) includes chemotherapy, radiotherapy and surgery [1, 2], side-effects of which are wide-ranging physical deficits [3–6]. Functional outcome assessments are crucial to assess the impact of treatment as well as the response to rehabilitation interventions [7]. Established sarcoma assessments including; a disability-specific questionnaire ‘Toronto Extremity Salvage Scale (TESS)’ comprising of 30 questions about the difficult in performing activities of daily living [4] and a clinician-reported physical impairment tool ‘Musculoskeletal tumour rating system (MSTS) [8, 9] comprising of 7 items such as range of motion (ROM), muscle strength, pain, functional activity, joint instability, deformity and emotional acceptance of the limb have inherent limitations [7]. For instance: these do not capture important objective information about postural control (balance), gait and movements related to activities of daily living [5, 10]. Clinical tools for assessing postural control and movement range from simple, time-based assessments through to full-body kinematic and kinetic examinations [11]. Simple assessments provide useful information about body stability in space, but are prone to ceiling effects and may not accurately quantify postural control strategies [11, 12].

Adding more advanced data collection tools such as force platforms and three-dimensional (3D) camera systems allows for a more detailed analysis. In addition to measuring limb movement during a functional reach test, a 3D camera system can capture spatiotemporal measures of movement trajectories and kinematics [13]. This could be particularly useful in determining patient-specific movement and stability techniques which could uncover maladaptive strategies for maintaining balance and movement [14]. A major disadvantage of these systems however is that they require multiple cameras, a large space and skin-based markers. They are also cumbersome to house and transport, expensive and need technical expertise to use and interpret. These factors limit their use to major clinical centers and research laboratories.

Markerless motion capture (MMC) can overcome limitations of traditional systems as they have the potential to remove the possible influences of body-mounted markers on people and can also promote assessments both in the clinic and natural environments [15–18]. MMC using depth-sensors have gained popularity due to advantages such as, (1) are inexpensive compared to expensive laboratory systems like the Vicon 3D motion capture, (2) are portable and (3) can perform accurate 3D tracking [19–21]. One such sensor been used more over recent years to perform MMC is the Microsoft Kinect^TM^ (Kinect Version 1) device, developed for gaming with the Microsoft Xbox One console [19]. Kinect uses video and infra-red cameras to create a 3D map of the capture volume [19]. An inbuilt randomised decision forest algorithm automatically determines anatomical landmarks on the body, including joint centers, in close to real time [22]. In the past decade, there has been growing interest in using the Kinect for general purpose motion capturing (MoCap) [23, 24]. It has shown fair to good accuracy in assessing; postural control [21], functional movements [15], 3D position in a workplace environment [16] and classifying dance gestures when compared to gold standard assessments. Furthermore, other areas where depth sensors have been used are for indoor depth mapping applications [19]. Kinect has been used for functional movement analysis including the assessments of spatial-temporal and kinematic variables of gait and staircase ascent and descent against gold standard assessments of traditional marker-based Motion Analysis systems like Vicon 3D motion capture [23–27]. As Kinect has shown promising results in the literature, in this study, the depth-sensor ‘Microsoft Kinect™’ (Kinect v1) was therefore selected as the technology of choice to perform MMC.

The overall objective of this study therefore was to establish the feasibility and explore early indicators of validity of inexpensive 3D MMC to measure clinically relevant movement in patients treated for lower extremity musculoskeletal cancer. Specific research questions were: Does MMC (1) demonstrate feasibility of use and acceptability [28] in a clinical setting? (2) produce clinically useful data comparable to the literature (an indicator of face validity) [29] (3) distinguish between tumour sub-groups (an indicator of discriminant validity) [30] and (4) relate to existing clinical measures (an indicator of convergent validity) [30] and standard manual techniques (an indicator of concurrent validity) [31]?

## 2. Materials and methods

### 2.1 Patient group

The study was approved by the National Research Ethics committee (Reference: 13/NE/0296) and the Newcastle Upon Tyne Hospitals NHS Foundation Trust, R&D department (Reference: 6801).As this study involves participation by human research participants, the data sharing is therefore bound by the National Research Ethics Committee (NREC) approval for the study which we need to abide by.

Eligible patients were approached and recruited using convenience sampling from clinics and databases between 01.02.2014 and 31.10.2014. The data was accessed for research purposes between 31.10.2014 and 21.07.2021 for processing and analysing the raw data, conducting statistical analysis and dissemination purposes e.g. writing-up reports, presenting at conferences and submitting to journals. After successfully publishing two key papers from this large project, on the topics of the use of triaxial accelerometer-based body worn monitor technology in the clinic [10] and in the community [32] respectively; this current paper which investigated a different dataset of MMC outcomes to answer questions about the feasibility and clinical applicability of the use of depth sensors for MMC was compiled. The current paper is different from the previous two publications, as it investigates the MMC approach which has never been done before for this cancer group.

Patients were enrolled into the study using Informed Consent. All patients provided written informed consent. Patients included were more than one-year post-surgery for a lower extremity musculoskeletal tumour and free of active disease or active treatment. As this was a pilot and feasibility study, the sample size established for the study was 40 patients as pilot studies recommend including over 30 patients [33]. The study included both children and adults but for the purpose of investigating MMC, 34 adult datasets were used for final analysis [33]. Our pilot study is a feasibility study exploring the feasibility of a new approach MMC for this cancer population and is of a descriptive nature, so can give an indication about what is going on with the applicability of this new approach for this patient group. We are not concerned about the representativeness of this pilot group using convenience sampling, as it is only giving us an idea about the feasibility of a new approach. The data collected and tested using statistical analysis will give us trends/early results or indicators for the next larger study which will be appropriately powered and this larger study will use probability sampling to test hypothesis rigorously. Additional details about the approach to sampling and recruitment can be found in a previous publication [10].

### 2.2 Study design

Cross Sectional Pilot and Feasibility Study.

### 2.3 Equipment

The MMC system, Microsoft Kinect™ is a motion sensor which captures functional movements in 3D space. The Kinect sensor version 1 includes skeletal tracking software which estimates the position of anatomical landmarks, such as joint centers, in close to real time [34] at a 30 Hz and 640 x 480 px spatial and depth resolution. Data were obtained from the Kinect using the Microsoft software development kit (SDK). Skeleton joint position data were obtained in three axes (converted to X = mediolateral, Y = anteroposterior, Z = vertical) from patients (Supplementary material: S1A and S1B Fig).

### 2.4 Tests performed

3D MMC Methodology: Participants stood facing the Kinect sensor at a distance of 3 metres (m), adequate to collect accurate data without causing interference [16]. The sensor was placed 1 m from the floor, with the lens perpendicular to the ground, pointing towards patients. The tests included: (1) the walk component of a 7-metre timed up and go (TUG) test, (2) unilateral stance (UL), (3a) stand to kneel (STK) and (3b) kneel to stand (KTS). A researcher stood beside the Kinect to demonstrate movements and in some instances closer to the patient to ensure their safety. Patients who underwent an amputation were offered bilateral support if they wished to perform the test on the prosthetic leg. For those who did not wish to perform the test on their prosthetic leg, their wishes were respected. Feedback forms were used to capture patient experiences about MMC using Kinect on acceptability, user-friendliness and comfort. Each patient completed a feedback form which asked whether patients found MMC acceptable (Yes/No), user-friendly (Yes/No) and comfortable (Yes/No). Patients were also given the opportunity to write open comments about their experience of the depth sensor.

### 2.5 Assessment using video

Temporal measures of STK and KTS test was calculated using observational video analysis and MMC. The start and finish of each activity was selected based on the start and the end of the movement of the head. Video derived time was not selected for other tests (Unilateral stance and TUG test) because the temporal measures captured during these tests were not indicative of the time taken to complete the activity. On the other hand, temporal measures of KTS and STK indicated the time taken to complete kneeling and rising from kneeling activity. MMC versus Video was therefore compared for only STK and KTS activities, to explore early indicators of concurrent validity.

### 2.6 Tumour sub-groups

Patients were grouped by tumour type [bone tumour (BT) or soft tissue sarcoma (STS)] and surgery [limb sparing (LSS) or amputation (AMP)]. MMC outcomes were compared between tumour groups to explore early indicators of discriminant construct validity [30].

### 2.7 Clinic scales in sarcoma

Traditional sarcoma measures; disability (TESS) [4], impairment (MSTS) [8, 9], quality of life (Quality of life-Cancer survivors (QoL-CS) [35] (Table 1) were collected. Clinical scores were collected to compare validated clinical tools in the sarcoma population (e.g TESS) against MMC; to investigate whether clinically sensible relationships were observed between the measures. This was assessed using the ICF biopsychosocial health model [36, 37] to explore whether sensible clinically sensible relationships existed, to explore early indicators of convergent construct validity [30].

**Table 1:** Existing clinical measures and MMC measures [obtained using Microsoft Kinect^TM^] for patients with tumours.

S.No	Clinic measures	Sub-domains/What does the outcome measure capture	Outcomes	Scores
***Existing clinic measure***				
1.	Toronto Extremity Salvage Score (TESS) (4)	30-item reported by patients	Physical disability	Scores range from 0 to 100 (worst to best outcomes)
2.	Quality of Life for Cancer Survivors (QoL-CS) (23)	41-item questionnaire	QoL	Scores range from 0 to 100 (worst to best outcomes)
3.	Musculoskeletal Tumour Society score (MSTS) version developed in 1987 (MSTS-1987) for the Lower Limb (8)	7 sub-domains range of motion, stability, deformity, pain, muscle strength, functional activity and emotional acceptance	Impairment	The MSTS total score is expressed from 0–35 (worst to best physical functioning). Individual sub-domain score is 0–5
***Outcome measures derived from MMC***				
*Test 1*: Walk component of Timed Up and Go Test (TUG) (Repeat x3): Patients were asked to complete a timed up and go test with Kinect recording the walking component of test. Horizontal displacement of the head marker was used to derive outcome measures				
1.	Walk Distance (m)	Walk distance recorded by MMC	Spatial gait	A lower walk distance compared to healthy individuals reflects impaired gait
1.	Walk Time (s)	Walk time to cover the distance	Temporal gait	A higher walk time compared to healthy individuals reflects impaired gait
2.	Walk Velocity (m/s)	Walk velocity during this distance	Spatio-temoral gait	A lower walk velocity compared to healthy individuals reflects impaired gait
*Test 2*: Unilateral stance for up to 30 seconds on affected and unaffected limb (Repeat x3): Patients started from a position of standing with eyes open. Patient was asked to lift one leg off the floor without support if able. If patient was unable to perform the test without support, the test was performed with support and this was recorded. Vertical displacement of the knee marker was used to identify the start and end of the test and the movement of the head marker was utilised to derive outcome measures				
1.	Unilateral stance total time (s)	Time for which patients’ could perform the unilateral stance test	Temporal measure	A lesser time compared to healthy individuals reflects impaired balance [38]
2.	Unilateral stance Anterior-posterior range (m)	Distance a patient swayed in the AP direction during their unilateral stance test	A-P Balance	A higher range of sway compared to healthy individuals reflects impaired balance [21]
3.	Unilateral stance Medio-lateral range (m)	Distance a patient swayed in the ML direction during the unilateral stance test	M-L Balance	A higher range of sway compared to healthy individuals reflects impaired balance [21]
3.	Unilateral stance Antero-posterior sd (m)	Standard deviation (sd) of change of AP range	A-P Balance change	A higher range of sd compared to healthy individuals reflects impaired balance [21]
4.	Unilateral stance Medio-lateral sd (m)	Standard deviation (sd) of change of ML range	M-L Balance change	A higher range of sd compared to healthy individuals reflects impaired balance [21]
*Test 3a*: Stand to kneel test (STK, Kneeling Activity) (Repeat x3): Patients started the test from a position of standing with eyes open. Patients were asked to kneel on a firm kneeling mat without support where able. Vertical displacement of the head marker was used to derive outcome measures				
1.	STK Total time (s)	Time taken to perform stand to kneel test	Temporal measure	The ability to complete kneeling is a good outcome [39]. However a higher total time compared to healthy individuals reflects an impaired activity [39]
2.	STK Peak velocity (m/s)	Maximum velocity attained during stand to kneel test	Spatio-temporal	A lower peak velocity compared to healthy individuals reflects poor balance/postural control during kneeling [40]
3.	STK Anterior amplitude (m)	Maximum anterior amplitude attained during kneeling activity	A-P Balance	Similar to unilateral stance reasoning [21], a higher amplitude of sway compared to healthy individuals reflects an impaired balance/postural control during kneeling.
4.	STK Lateral amplitude (m)	Maximum lateral amplitude attained during kneeling activity	M-L Balance	A higher amplitude of sway compared to healthy individuals reflects an impaired balance/postural control during kneeling, as the participant is moving out of their base of support.
*Test 3b*: Kneel to stand test (KTS, Rising from Kneeling Activity) (Repeat x3): Patients started the test from a position of kneeling. Patients were asked to stand from kneeling, without support where able. Support was provided when required and this was recorded for Tests 3. Vertical displacement of the head marker was used to derive outcome measures				
1.	KTS Total time (s)	Time taken to perform kneel to stand test	Temporal measure	A higher total time compared to healthy individuals reflects an impaired activity
2.	KTS Peak velocity (m/s)	Maximum velocity attained during rising from kneeling activity	Spatio-temporal measure	A lower peak velocity compared to healthy individuals reflects poor balance/postural control during rising from kneeling [40]
3.	KTS Anterior amplitude (m)	Maximum anterior amplitude attained during kneel to stand test	A-P Balance	A higher amplitude of sway compared to healthy individuals reflects an impaired balance/postural control during kneeling
4.	KTS Lateral amplitude (m)	Maximum anterior amplitude attained during during kneel to stand test	M-L Balance	A higher amplitude of sway compared to healthy individuals reflects an impaired balance/postural control during kneeling.

### 2.8 Processing of data obtained from MMC

Raw MMC data was screened and activity-stamped visually using Microsoft Excel 2013 (v15.0). Following this, data processing used anatomical landmark displacements during tests. Raw data from walk, unilateral stance, STK and KTS (Supplementary material: S2A–S2C Fig) were processed to obtain MMC measures by activity (Table 1, Supplementary material: S2A–S2C Fig). Outcome measures obtained from MMC were then classified by known functional domains as follows [15, 21, 24–26] and used for final analysis.

**(a) Temporal:** Time-based measures recorded during unilateral stance, STK and KTS performance measured in seconds

**(b) Balance:** Amplitude of shift from midline and standard deviation were used as balance outcomes keeping with standard clinical outcomes. Balance outcomes were collected during the unilateral stance, STK and KTS tests measured in metres (m). During unilateral stance, the knee marker was used to capture the start and the end of the test and the movement of the head marker was utilised to measure postural sway. For the STK and KTS tests, the movement of the head marker was used to determine the extent of postural sway from midline.

**(c) Gait:** Walking distance, time and velocity obtained from walk component of 7-metre TUG test and was measured in m, s and m/s.

**(d) Movement velocity:** Movement velocities recorded during STK and KTS measured in m/s^.^ Movement velocity is defined as vertical velocity. The start point and the end point were defined by the movement of the head from kneeling to stand and stand to kneel.

### 2.9 Clinical interpretation of good versus poor clinical outcomes

The clinical interpretation of poor and good outcomes have been detailed in Table 1.

### 2.10 Skeletal model tracking

The MMC system, Kinect SDKs provide a skeleton tracker of up to 20 body joints and code samples that can be used to track movement. Patient movements can be visualised without using video. The skeleton tracker classifies each pixel of depth images as components of a joint using trained decision forests [22].

### 2.11 Statistical analysis

Statistical analysis was carried out using SPSS v19 (IBM). Parametric data were expressed using means and standard deviations (SDs) (minimum value—maximum value) and non-parametric data using medians with interquartile ranges (IQR). Outcomes obtained using MMC were compared between tumour sub-groups using Independent t or Mann-Whitney U tests. We used the Bonferroni correction to address correction for multiple measures for the between group comparisons and set the alpha level at 0.05/6 = 0.008. Pearson and Spearman’s rho correlations were used to investigate relationships between measures obtained from MMC and existing measures. Intraclass correlation coefficient (ICC) agreement, two-way random effects model and Bland Altman analysis tested agreement between MMC measures and standard manual techniques. ICC, is a descriptive statistic which was used to describe the strength of units in the same group and their resemblance with each other. We interpreted ICC agreements as: poor (< 0.5), moderate (between 0.5 and 0.75), good (0.75 to 0.9) and excellent (> 0.9) (30, 31). Significance was taken at the 0.05 level.

## 3. Results

### 3.1 Demographic and clinical characteristics

34 adults of mean age 43 ± 20 years participated. Patients were treated for BT (n = 21) or STS (n = 13) in the femur (n = 19), pelvis/hip (n = 3), tibia (n = 9), or ankle/foot (n = 3). 27 underwent LSS and 7 AMP. Median time from surgery was 79 months (33–108). 15/34 patients received chemotherapy, and 13/34 received radiotherapy. Further details about potentially eligible patients approached, those who refused and participated can be found in the recent publication [10]. A total of 97 patients were approached in this study, 65 patients satisfied all eligibility criteria and 32 patients were ineligible. Out of the 65 patients, 21 patients declined participation and 4 patients were non-contactable, leaving us with 40 patients in the study out of which 34 were adults and their data were used for final analysis. Patients who declined participation were mainly those who lived far away from the specialist centre where assessments were undertaken and did not prefer to travel these distances to participate in the research.

### 3.2 Feasibility, data loss and acceptability of MMC in the clinic

MMC was feasible to perform using Kinect and was quick to set up, taking approximately 10 minutes. Data downloading and processing were straightforward taking approximately 20 minutes to complete. Of 34 participants, one had a hind quarter amputation and could not perform any clinic tests. Furthermore, the MMC system did not record data for 3 patients due to a technical issue with data collection. Of the remainder, 8 were either unable to or refused to perform kneeling and 2 unilateral stance on the affected side. During data processing, 2 trials data (one kneeling and one unilateral stance) were invalid and removed from final analysis. Therefore of 33 patients assessed, data related to 30 walking, 21 kneeling, 27 unilateral stance on affected side and 30 unilateral stance on unaffected side test datasets were available for analysis.

Of patients who returned feedback forms with questions answered about the MMC system (n = 19), patients who found the MMC approach in the clinic: acceptable for use: 19/19 (100%) and comfortable: 19/19 (100%) after sarcoma treatment.

### 3.3 Early Indicators of Validity of MMC in musculoskeletal cancer patients

Outcome measures derived from MMC are summarised in Table 2.

**Table 2:** Functional outcomes in tumour patients captured using MMC, BT vs STS, LSS vs AMP.

Outcome measures derived from MMC	Tumour patients (n = 33)	BT group (n = 21)	STS group (n = 12)	p-value for BT vs STS groups	LSS group (n = 27)	AMP group (n = 6)	p-value for LSS vs AMP groups
	Median/Mean Values (25^th^– 75^th^ percentile, 1QR/Min -max)	Median/Mean Values (25^th^– 75^th^ percentile, 1QR/Min -max)	Median/Mean Values (25^th^– 75^th^ percentile, 1QR/Min -max)		Median/Mean Values (25^th^– 75^th^ percentile, 1QR/Min -max)	Median/Mean Values (25^th^– 75^th^ percentile, 1QR/Min -max)	
**Temporal measures (assessing time taken to complete/sustain activity)**							
Unilateral stance total time (s) of affected limb	20.38	20.79	11.77	0.111	20.53	3.96	0.382
	(6.81–29.32)	(8.27–29.46)	(3.82–18.68)		(8.27–27.62)	(1.81–29.56)	
Unilateral stance total time (s) of unaffected limb	29.22	29.69	26.61	0.047*	29.36	29.04	0.795
	(22.71–29.92)	(25.56–30.00)	(15.88–29.49)		(21.40–29.92)	(19.30–29.86)	
STK total time (s)	2.00	2.05	1.90	0.881	1.90	2.24	0.585
	(1.48–2.82)	(1.51–2.81)	(1.430–3.80)		(1.53–2.83)	(1.43–3.40)	
KTS total time (s)	2.83	3.37	2.53	0.551	2.67	3.37	0.586
	(1.90–3.84)	(2.06–3.82)	(1.53–4.83)		(1.77–3.80)	(2.33–4.26)	
**Balance measures (assessing the range during movements or high amplitude of shift from midline or base of support)**							
Unilateral stance A-P range (m) of affected side	0.08	0.07	0.11	0.088	0.07	0.13	0.177
	(0.05–0.11)	(0.05–0.10)	(0.06–0.21)		(0.05–0.10)	(0.06–0.20)	
Unilateral stance M-L range (m) of affected side	0.06	0.06	0.08	0.059	0.07	0.06	0.706
	(0.05–0.09)	(0.04–0.07)	(0.07–0.10)		(0.05–0.08)	(0.04–0.20)	
Unilateral stance A-P sd (m) of affected side	0.02	0.02	0.02	0.054	0.02	0.02	0.307
	(0.01–0.02)	(0.01–0.02)	(0.02–0.06)		(0.01–0.02)	(0.01–0.06)	
Unilateral stance M-L sd (m) of affected side	0.02	0.01	0.02	0.064	0.02	0.02	0.314
	(0.01–0.02)	(0.01–0.02)	(0.02–0.03)		(0.01–0.02)	(0.01–0.06)	
Unilateral stance A-P range (m) of unaffected side	0.08	0.08	0.08	0.564	0.08	0.13	0.006*
	(0.06–0.12)	(0.06–0.12)	(0.07–0.14)		(0.06–0.11)	(0.11–0.21)	
Unilateral stance M-L range (m) of unaffected side	0.05	0.05	0.05	0.875	0.05	0.07	0.021*
	(0.04–0.07)	(0.04–0.07)	(0.04–0.07)		(0.04–0.07)	(0.05–0.16)	
Unilateral stance A-P sd (m) of unaffected side	0.02	0.02	0.02	0.963	0.01	0.03	0.004*
	(0.01–0.02)	(0.01–0.03)	(0.01–0.02)		(0.01–0.02)	(0.02–0.04)	
Unilateral stance M-L sd (m) of unaffected side	0.01	0.01	0.01	0.476	0.01	0.02	0.014*
	(0.01–0.02)	(0.01–0.02)	(0.01–0.01)		(0.01–0.01)	(0.01–0.03)	
STK Anterior amplitude (m)	0.22	0.14	0.31	0.048	0.22	0.23	0.533
	(0.11–0.31)	(0.10–0.28)	(0.22–0.44)		(0.08–0.31)	(0.14–0.34)	
STK Lateral amplitude (m)	0.17	0.18	0.12	0.295	0.12	0.27	0.507
	(0.05–0.32)	(0.07–0.39)	(0.03–0.26)		(0.05–0.26)	(0.04–0.41)	
KTS Anterior amplitude (m)	0.13	0.13	0.12	0.764	0.12	0.16	0.079
	(0.10–0.24)	(0.10–0.25)	(0.08–0.25)		(0.08–0.23)	(0.13–0.34)	
KTS Lateral amplitude (m)	0.10	0.11	0.10	0.572	0.12	0.02	0.813
	(0.02–0.18)	(0.01–0.28)	(0.01–0.17)		(0.01–0.17)	(0.02–0.37)	
**(C) Gait measures (assessing parameters of gait)**							
Walk distance (m)	2.50	2.52	2.43	0.481	2.48	2.60	0.392
	(2.21–2.77)	(2.19–2.8)	(2.21–2.65)		(2.18–2.73)	(2.03–2.99)	
Walk time (s)	2.24	2.28	2.12	0.244	2.24	2.27	0.917
	(1.84–2.57)	(1.8525–2.6475)	(1.79–2.39)		(1.85–2.56)	(1.71–2.93)	
Walk velocity (m/s)	1.10	1.08	1.26	0.058	1.09	1.13	0.959
	(0.89–1.30)	(0.80–1.14)	(1.03–1.36)		(0.91–1.31)	(0.75–1.23)	
**(D) Movement velocity measures (assessing the velocity during functional movements)**							
STK Peak velocity (m/s)	-0.57	-0.59	-0.54	0.278	-0.57	-0.59	0.696
	[(-0.62-(0.44)]	[(-0.72-(0.42)]	[(-0.57–0.44)]		[(-0.63 - (-0.44)]	[(-0.70 –(-0.42)]	
KTS peak velocity (m/s)	0.82	0.84	-0.54	0.247	0.68	0.93	0.959
	(0.61–0.99)	(0.63–1.05)	[(-0.57- (-0.44)]		(0.55–0.88)	(0.71–1.14)	

Statistical test: Mann-Whitney U test. p-value–correlation between variables (* = statistically significant with and without Bonferroni correction

#### 3.3.1. Outcomes from MMC in tumour sub-groups

The MMC approach distinguished between BT and STS groups and the LSS and AMP groups for temporal and balance outcomes respectively in the unaffected limb (p<0.05*) (Table 2). For instance, the Unilateral Stance total time (s) of unaffected limb was found to be higher in patients operated for bone tumours 29.69 (25.56–30.00) s than those operated for soft tissue tumours 26.61 (15.88–29.49) s, indicating that patients with bone tumours could stand on their unaffected limb for longer than those with soft tissue tumours. Furthermore the Unilateral stance A-P range (m) and M-L range (m) of unaffected side and the Unilateral stance A-P sd (m) and M-L of unaffected side were found to be significantly higher in patients who had an amputation compared to those with limb sparing surgeries (p<0.05*) (Table 2).

#### 3.3.2. Outcome measures from MMC versus clinical scales

**Fig 1:**
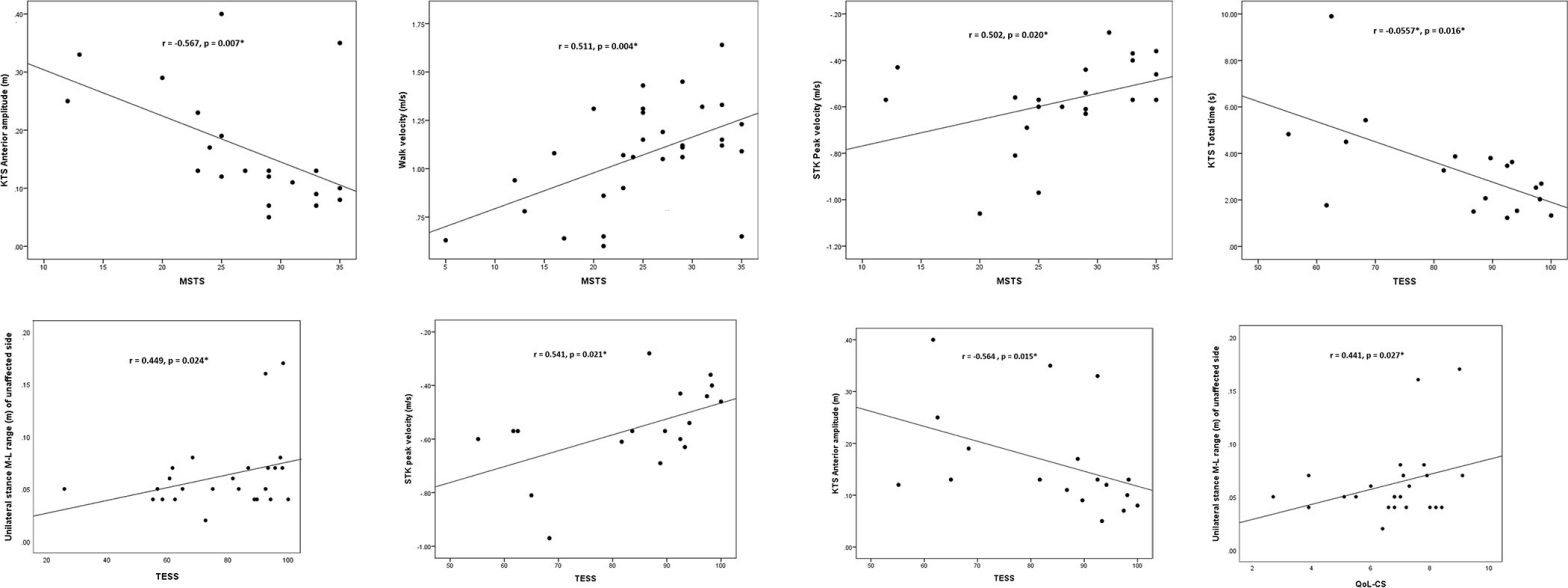
Correlations of traditional sarcoma measures with outcomes captured by MMC. Outcomes captured by MMC significantly relate to MSTS, TESS and QoL-CS (Fig 1A–1H). A: KTS Anterior amplitude significantly relates to MSTS. B: Walk velocity significantly relates to MSTS. C: STK Peak velocity significantly relates to MSTS. D:KTS Total time relates to TESS. E:Unilateral stance M-L range of unaffected side relates to TESS. F:STK Peak velocity relates to TESS. G:KTS Anterior amplitude relates to TESS. H: Unilateral stance M-L Range of unaffected side relates to QoL-CS.

Median (range) TESS score was 83.6 (IQR 62.1 to 93.8 [8.3 to 100.0]), mean MSTS score 24.5 (SD 7.9 [5.0 to 35.0]), median 3-meter TUG time 10.8 seconds (IQR 8.5 to 12.7 [7.9 to 32.3]) and median QoL-CS total score 7.1 (IQR 6.1 to 7.8 [2.7 to 9.1]). Significant correlations were observed between MSTS, TESS, QoL-CS and MMC (p<0.05*) (Table 3 and Fig 1A–1H).

**Table 3:** Relationships between established clinical scales and outcome measures derived from MMC.

Clinical scales in sarcoma	(A) Temporal measures	r value	p-value	(B) Balance measures	r value	p-value	(C) Gait Measures	r value	p-value	(D) Movement velocity measures	r value	p-value
MSTS total (impairment)	Unilateral stance of affected side total time (s)	0.268	0.176	KTS Anterior amplitude (m)	-0.567	0.007*	Walk distance (m)	0.005	0.978	STK peak velocity (m/s)	0.502	0.020*
	STK Total time (s)	-0.206	0.370	Unilateral stance A-P range of affected side	0.064	0.751	Walk time (s)	-0.416	0.022*	KTS peak velocity (m/s)	-0.030	0.896
	KTS Total time (s)	-0.347	0.124	Unilateral stance M-L range of affected side (m)	0.140	0.487	Walk velocity (m/s)	0.511	0.004*			
TESS (disability)	Unilateral stance total time (s)	0.340	0.113	Unilateral stance M-L range of unaffected side (m)	0.449	0.024*	Walk distance (m)	-0.045	0.831	STK peak velocity (m/s)	0.541	0.021*
	STK Total time (s)	-0.372	0.128	KTS Anterior amplitude (m)	-0.564	0.015*	Walk time (s)	-0.237	0.253	KTS peak velocity (m/s)	-0.011	0.964
	KTS Total time (s)	-.0557*	0.016*	STK Anterior amplitude (m)	0.236	0.345	Walk velocity (m/s)	0.291	0.159			
QoL-CS total score	Unilateral stance total time (s)	0.130	0.554	Unilateral stance M-L range of unaffected side (m)	0.441	0.027*	Walk distance (m)	0.138	0.511	STK peak velocity (m/s)	0.243	0.331
	STK Total time (s)	-0.449	0.062	STK Anterior amplitude (m)	0.098	0.699	Walk time (s)	-0.162	0.439	KTS peak velocity (m/s)	-0.124	0.625
	KTS Total time (s)	-0.341	0.166	STK Lateral amplitude (m)	-0.433	0.072	Walk velocity (m/s)	0.358	0.079			
QoL-CS social sub-score (QoL)	STK Total Time (S)	-0.516	0.028*	KTS Anterior amplitude (m)	-0.492	0.038*						
	KTS Total Time (S)	-0.560	0.016*	KTS Lateral amplitude (m)	-0.522	0.026*						

Statistical test = Spearman’s correlation.

*A p value < 0.05 was considered statistically significant; BWM = body-worn monitor, TUG = timed up and go test; MSTS = Musculoskeletal Tumour Society Scoring system; TESS = Toronto Extremity Salvage Score; QoL-CS = Quality of life-Cancer survivors; RMS = Root Mean Square

For instance, MSTS demonstrated significant negative correlations with KTS Anterior amplitude. This suggested that high impairments of function i.e. impacted joint ROM, muscle strength, joint stability, limb length discrepancy, pain and gait problems (indicating high functional impairments) were associated with a large postural sway during kneeling (indicating affected balance). MSTS also correlated positively with STK peak velocity and walk velocity; suggesting high MSTS scores (low functional impairments) are associated with high movement velocity (good speed/function) during kneeling and higher walk velocity (good gait speed/function) respectively.

TESS showed significant negative correlations with KTS total time and KTS anterior amplitude, suggesting high levels of disability (low TESS) were associated with more time to execute KTS (poor function) and a high KTS anterior amplitude i.e. larger postural sway (indicating affected balance) during kneel to stand activity. TESS also showed significant positive correlations with Unilateral stance M-L range of unaffected side and STK peak velocity, suggesting that high levels of disability (low TESS) are associated with a low postural sway of the unaffected side during unilateral stance (good function on the unaffected side) and low movement velocity during STK (poor function), reflecting important associations.

QoL-CS showed significant positive correlations with Unilateral stance M-L range of unaffected side, suggesting that a reduced postural sway (impaired balance) on the unaffected side is associated with a reduced QoL. Whereas the QoL social sub-scales showed significant negative correlations with KTS Anterior amplitude and KTS Lateral amplitude. This suggested that larger posture sways in the anterior and lateral directions during KTS were associated with a poor QoL from a social integration perspective.

#### 3.3.3. Agreement of MMC with video for temporal accuracy

**Fig 2:**
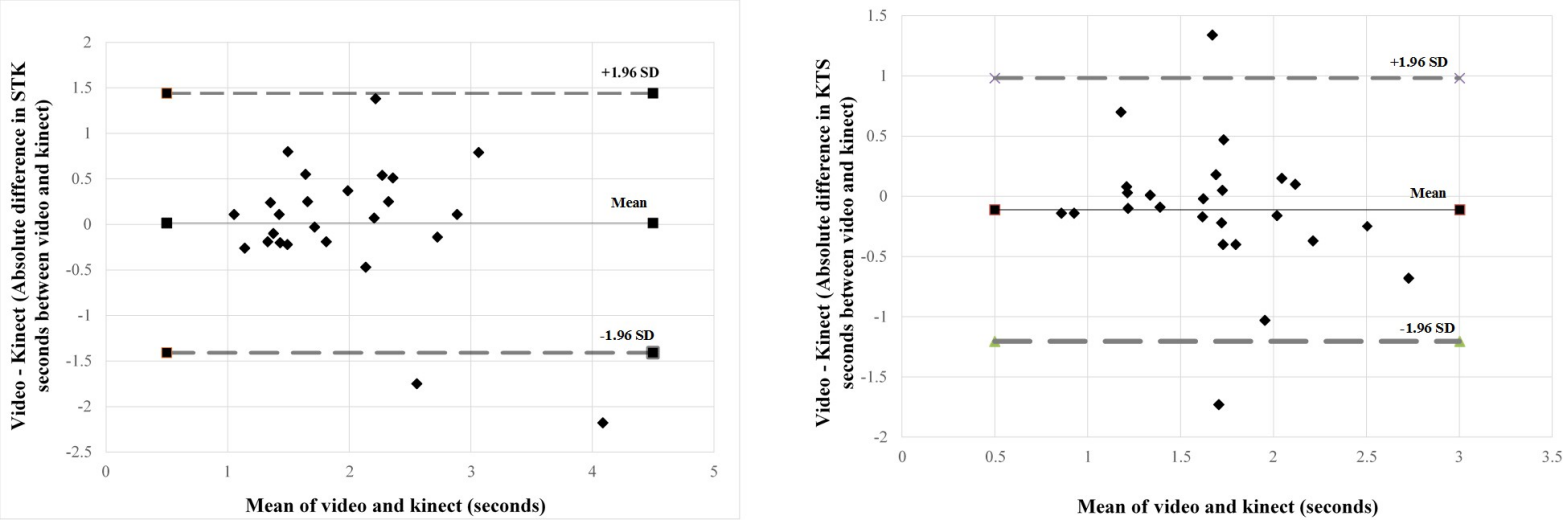
A: Agreement of Kinect and video times for STK. B: Agreement of Kinect and video times for KTS.

STK time captured by video [2.00 +/- 0.67 seconds] showed moderate agreement with STK time captured by Kinect [1.98+/- 0.90 seconds] (ICC agreement = 0.742; p = 0.001*) (Table 4). Bland-Altman analysis (Fig 2A) demonstrated that Kinect underestimated values with a bias of 0.014 in comparison to the video. The 95% limits of agreement were +1.44 seconds and -1.41 seconds.

**Table 4:** ICC agreement for STK time captured by video and MMC.

Test (n = 25)	Mean	SD	ICC average measures	95% confidence interval		p value
				Lower bound	Upper bound	
Kinect_STK_time(s)	1.98	0.90	0.74	0.41	0.89	0.001*
Video_STK_time(s)	2.00	0.67				

Cronbach’s alpha = 0.734

Cronbach’s alpha based on standardized items = 0.754

p value–agreement between devices (*statistically significant); two-way random effects model where both people effects and measures effects are random; ^†^the estimator is the same, whether the interaction effect is present or not; ^‡^type A intraclass correlation coefficients using an absolute agreement definition; ICC = intraclass correlation coefficients.

Similarly, KTS time captured by Kinect [1.73 +/- 0.60] (ICC agreement 0.624; p = 0.010*) showed moderate agreement with KTS time by video [1.62 +/- 0.47] (Table 5). Bland-Altman analysis (Fig 2B) demonstrated that Kinect over-estimated the values with a bias of -0.112 in comparison to video, with 95% limits of agreement of +0.98 seconds and -1.21 seconds.

**Table 5:** ICC agreement for KTS time captured by video and MMC.

Test (n = 25)	Mean	SD	ICC average measures	95% confidence interval		p value
				Lower bound	Upper bound	
Kinect_STK_time(s)	1.73	0.59	0.62	0.16	0.83	0.010*
Video_STK_time(s)	1.62	0.47				

Cronbach’s alpha = 0.624

Cronbach’s alpha based on standardized items = 0.635

p value–agreement between devices (*statistically significant); two-way random effects model where both people effects and measures effects are random; †the estimator is the same, whether the interaction effect is present or not; ‡type A intraclass correlation coefficients using an absolute agreement definition; ICC = intraclass correlation coefficients

#### 3.3.4. Case studies of skeletal model tracking

Skeletal tracking data captured for Case study A during the unilateral stance test and Case study B during the STK and KTS test (Supplementary material: S1 Table) demonstrated that it is feasible to collect and visually inspect movements or activities from anonymised data collected; in patients with sarcomas.

## 4. Discussion

### 4.1. Feasibility and acceptability of MMC

This is the first pilot to investigate MMC in patients with musculoskeletal cancers using a low-cost portable depth-sensor. Overall, the MMC approach was found to be feasible to deliver, straightforward to collect and acceptable to use and comfortable by those who completed the feedback survey. MMC also confirmed early indicators of validity for certain measures and sensible clinical trends were present in others which was promising. The positives were that MMC captured temporal measures, balance, gait and movement velocities outcomes promptly, with minimal data loss. MMC data could also assess slowness of activities in patients, postural control strategies, gait and movement velocities alterations, not obtainable routinely but which add novel knowledge to guide rehabilitation and treatment after major surgery for this rare cancer.

### 4.2. Early Indicators of validity of MMC

#### 4.2.1. Comparison to healthy individuals from reference literature

Outcomes derived from MMC made broad clinical sense and some variables were comparable to published literature [21, 24]. Unilateral stance anterior-posterior (A-P) range (m) of the affected side [0.08 (0.05–0.11) m] and, unilateral stance medio-lateral (M-L) range (m) of affected side [0.06 (0.05–0.09) m], were higher than balance values reported by Clark et al for healthy individuals [0.051 (0.032) m] [21]. The closest anatomical marker in Clark’s study which compared to our study’s head marker was the sternum marker assessing balance in unilateral stance. Although there was a difference in the protocol of both studies, patients treated with sarcoma presented with a higher postural sway in the A-P direction while standing on their affected limb [0.08 (0.05–0.11) m] compared to healthy individuals [0.06 (0.02) m] in Clark’s study. This demonstrates that patients treated with sarcoma demonstrate reduced postural control than healthy individuals putting them at a higher risk of falls, which can be attributed to postural control changes in the affected side after major surgical resections. A-P sway was higher compared to M-L sway which highlight the postural compensatory mechanisms in people operated for sarcomas.

In previous studies, balance and postural control strategies during quiet standing were found to be affected in patients treated for sarcoma [5, 10] however postural control strategies during other activities were never investigated. This is the first study demonstrating that postural control strategies measured objectively are affected during unilateral stance and kneeling and require targeted rehabilitation e.g. balance exercises to improve postural control during such activities.

Similarly for gait outcomes, our study patients showed gait velocity [1.10 (0.89–1.30) m/s] was lower than the mean gait velocity [1.26±0.12 m/s] in healthy adults from another study [24]. This could be explained on the basis that our study patients had large surgeries on their lower limbs which affect locomotion and gait.

#### 4.2.2. Comparison between tumour sub-groups

In our study, the MMC approach distinguished between BT and STS groups, and these comparisons were made as BT are often deep seated compared to STS which are usually more superficial and this often clinically results in differences between BT and STS groups. However as this was pilot and feasibility work, the extent of surgery and reconstruction were not collected. In future work, this will be considered as the size of the tumour and extent of surgery plays a big role alongside the primary diagnoses of the disease. For example, the functional outcome may be the same in large sized sarcomas whether primarily affecting bone or soft tissues.

MMC distinguished between LSS and AMP groups for temporal and balance outcomes in the unaffected limb (p<0.05*). Amputees demonstrated a significantly higher unilateral stance A-P range (m) and M-L range (m) of the unaffected side compared to the LSS group (p<0.05). This could be explained on the basis of alterations in the sensory and proprioceptive inputs after a major limb loss [41, 42] potentially leading to significant alterations and compensations in the unaffected side. This suggests that unaffected side testing is a sensitive test for patient groups.

#### 4.2.3. Outcome measures derived from MMC versus clinical scales

Our study revealed important associations between MMC and established clinical measures. Higher impairment was associated with poor balance, gait and movement velocity, which in turn, were significantly associated with worse disability and QoL. This emphasises the importance of using MMC, as findings revealed transition to and from floor activities, inherently known to be challenging for this patient group [3, 4, 43], is associated with a poor QoL. This novel information highlights relationships between outcomes and can guide the delivery of rehabilitation. For instance, in order to improve QoL of patients, people’s impairments such as joint range of motion, muscle strength, balance and gait are important to treat using targeted exercise prescriptions. MMC alongside clinical scores provide sensible relationships and comprehensive information about the patient’s functional status which is not traditionally captured.

#### 4.2.4. Agreement of MMC with manual techniques

Galna et al in 2014 reported that MMC consistently over- or under-estimated some measures of spatial and temporal movements compared to a 3D marker-based motion analysis system, dependant on the specific movement [15]. This study in people was in people with Parkinson’s. Our results agree with this study, as MMC in our study underestimated the STK total time and overestimated the KTS and showed moderate agreements. Calibrations might address such biases from MMC systems, along with future software updates which might help in overcoming some of the observed differences. Addressing biases could minimise errors and facilitate MMC to become a promising clinical assessment for risk-based screening programs in clinics and homes of patients.

### 4.3. Strengths, limitations, clinical implications and future work

#### 4.3.1. Strengths

This study is invaluable as a first step, to represent the use of this novel approach in this patient group. This pilot work tested the applicability of MMC across a wide range of the heterogenous population and demonstrated, which represents generalisability to all sub-groups. Patients who responded to the feedback survey had positive feedback about MMC which was promising.

#### 4.3.2. Limitations

The authors are aware that production of the depth-sensor used in this study has stopped but as technology evolves newer systems for performing MMC will be available. An important message from this first study of its kind; is MMC feasibility and its agreement with standard clinical methods which when supported with future research can facilitate its translation into busy clinical settings. Although. we particularly used the head and knee anatomical landmark displacements, a more comprehensive approach to detail postural control strategies by Clark et al. would be helpful. This approach looks at multiple points such as pelvis center, hip, hand, elbow, shoulder, sternum, ankle displacement data streams to derive quantitative data [21], to assess adaptive mechanisms, postural reactions and compensations for a complex patient group of sarcomas.

Another limitation was that unilateral stance with eyes closed was not performed, and so we are unable to comment on whether MMC can detect adaptive mechanisms performed by patients to compensate when vision is removed. Despite two reminders sent to patients, the response rate for the feedback points was found to be only 50% (19/34). This can introduce bias about acceptability and user-friendliness of MMC and results need to be treated with caution. In a larger study, we will approach this issue by completing feedback forms with patients in clinic or on phone, rather than by post to ensure a good response rate is achieved.

Although this was a pilot sample of convenience and have its inherent limitations; the results of this study highlighted important results about feasibility. Being a pilot study, patient groups were required to be clubbed together to have sufficient numbers in sub-groups e.g. amputation and therefore interpretation about individual clinical groups e.g. above knee amputation or below knee amputation is not possible. This will be addressed in future work. where patients will be classified into homogenous groups for analysis and also healthy controls will be included for comparison. Use of convenient sampling is a weakness of our study, and the prevalence found in our study may differ from that in the full target population due to possible selection bias. To address this, for the larger future study more appropriate approaches to sampling e.g. purposive sampling will be used.

#### 4.3.3. Clinical implications

MMC can be used as a screening tool to identify patients with impaired balance, gait and movement control. Personalised balance and gait exercise programmes and introducing challenges like eyes closed, perturbations and changes in surfaces can improve movement restoration, agility and control of movements, preventing trips and falls, minimising injury, increasing confidence and reducing dependence; ultimately improving the QoL. The Skeletal tracking feature is useful for assessing movement quality and postural control strategies [21] in a way that effectively anonymises data and provides feedback to clinicians and patients to reduce movement compensations. The goal is to use such cost-effective systems as rehabilitation tools in this cancer group and explore its transferability across other cancers and orthopaedic surgery groups.

#### 4.3.4. Future work

The next steps would be to conduct a larger study with sufficient power using purposive sampling strategy to validate the MMC approach. In future, other methods such as stopwatch, wearables like accelerometery in the clinic [10] and community [32] or fitbits, IMUs, mobile apps or Xsens could be utilised to collect outcomes alongside MMC. Xsens will be a useful comparison to MMC, if joint range of motion is the area of focus. MMC, wearables and Vicon 3D motion capture could collect outcomes such as movement velocities, balance and temporal parameters, which can allow a cross validation work between portable cost-effective devices and Vicon 3D motion capture.

Another future step is that, the MMC protocols from this study could also be deployed in future larger multi-centre studies in sarcoma sub-groups for example, distal femoral replacements, proximal femoral replacements, pelvic resections, pelvic reconstructions to perform further gold standard validation work as described earlier. We aim to include an assessment of external validity within the next future multi-centre work to increase generalisability of the results. Using MMC systems e.g. the newer versions of Kinect with the Microsoft Xbox One console could be attractive if used for exergaming for this cancer population, especially popular amongst children and young people. We will also utilise MMC in future studies to remotely monitor falls and gait of patients at home [27, 44] and this could be particularly useful in global pandemic conditions e.g. during the recent covid-19, where remote and digital healthcare remains the best means to avoid transmission or spread of the virus and deliver better healthcare [45].

## 5. Conclusion

The MMC approach demonstrated feasibility and early indicators of certain types of validity in capturing novel information about temporal, balance, gait, movement velocity and skeletal tracking in patients treated for lower extremity musculoskeletal cancer. MMC moderately agreed with a clinically accepted standard assessment yet requires further work to overcome some technical limitations. Patients who responded with the feedback forms found MMC acceptable and comfortable but a larger study with a higher response rate is required to confirm these findings. Some of the MMC measures obtained in this study such as unilateral stance balance and temporal measures were able to discriminate between major tumour groups suggesting early indicators of discriminant validity. Furthermore, some MMC variables of temporal, balance, gait and movement velocity showed significant relationships with established sarcoma scales suggesting early indicators of clinical convergent validity. These findings and further validation work need to be undertaken in a future sufficiently powered study. This can further extend the use of MMC for clinical translation in busy clinics and in the community, to enhance rehabilitation practices in the sarcoma community.

## Supporting information

S1 Checklist STROBE statement—checklist of items that should be included in reports of observational studies. (DOCX)

S1 Fig A: A-P view of anatomical landmark detection using MMC. B: M-L view of anatomical landmark detection using MMC.
(TIF)

S2 Fig A: Raw MMC Data and Processed outcomes during walks of TUG test. B: Raw MMC Data and Processed outcomes during Unilateral Stance test. C: Raw MMC Data and Processed outcomes during Stand to Kneel and Kneel to Stand test.
(TIF)

S1 Table Skeletal tracking data. (PDF)

## References

[1] Gerrand C, Athanasou N, Brennan B, Grimer R, Judson I, Morland B, et al. UK guidelines for the management of bone sarcomas. Clinical Sarcoma Research. 2016;6(1):7. doi: 10.1186/s13569-016-0047-1 27148438 PMC4855334

[2] Dangoor A, Seddon B, Gerrand C, Grimer R, Whelan J, Judson I. UK guidelines for the management of soft tissue sarcomas. Clin Sarcoma Res. 2016;6:20. Epub 2016/11/29. doi: 10.1186/s13569-016-0060-4 ; PubMed Central PMCID: PMC5109663. 27891213 PMC5109663

[3] Davis AM, Punniyamoorthy S, Griffin AM, Wunder JS, Bell RS. Symptoms and their Relationship to Disability Following Treatment for Lower Extremity Tumours. Sarcoma. 1999;3(2):73–7. doi: 10.1080/13577149977677 . 18521266 PMC2395419

[4] Davis AM, Wright JG, Williams JI, Bombardier C, Griffin A, Bell RS. Development of a measure of physical function for patients with bone and soft tissue sarcoma. Qual Life Res. 1996;5(5):508–16. Epub 1996/10/01. doi: 10.1007/BF00540024 . 8973131

[5] Furtado S, Errington L, Godfrey A, Rochester L, Gerrand C. Objective clinical measurement of physical functioning after treatment for lower extremity sarcoma; A systematic review. Eur J Surg Oncol. 2016;43(6):968–93. doi: 10.1016/j.ejso.2016.10.002 27836415

[6] Rosenbaum D, Brandes M, Hardes J, Gosheger G, Rodl R. Physical activity levels after limb salvage surgery are not related to clinical scores-objective activity assessment in 22 patients after malignant bone tumor treatment with modular prostheses. Journal of surgical oncology. 2008;98(2):97–100. Epub 2008/06/04. doi: 10.1002/jso.21091 . 18521841

[7] Gerrand C, Furtado S. Issues of Survivorship and Rehabilitation in Soft Tissue Sarcoma. Clinical oncology (Royal College of Radiologists (Great Britain)). 2017;29(8):538–45. Epub 2017/04/27. doi: 10.1016/j.clon.2017.04.001 . 28442210

[8] Enneking WF. Limb Salvage in Musculoskeletal Oncology. New York (YK): Churchill Livingston; Modification of the system for functional evaluation in the surgical management of musculoskeletal tumors 1987:[626–39 pp.].

[9] Enneking WF, Dunham W, Gebhardt MC, Malawar M, Pritchard DJ. A system for the functional evaluation of reconstructive procedures after surgical treatment of tumors of the musculoskeletal system. Clinical orthopaedics and related research. 1993;(286):241–6. Epub 1993/01/01. 8425352

[10] Furtado S, Godfrey A, Del Din S, Rochester L, Gerrand C. Are Accelerometer-based Functional Outcome Assessments Feasible and Valid After Treatment for Lower Extremity Sarcomas? Clinical orthopaedics and related research. 2020;478(3):482–503. Epub 2019/08/08. doi: 10.1097/CORR.0000000000000883 ; PubMed Central PMCID: PMC7145056 Related Research® editors and board members are on file with the publication and can be viewed on request. 31390339 PMC7145056

[11] Visser JE, Carpenter MG, van der Kooij H, Bloem BR. The clinical utility of posturography. Clin Neurophysiol. 2008;119(11):2424–36. Epub 2008/09/16. doi: 10.1016/j.clinph.2008.07.220 . 18789756

[12] Blum L, Korner-Bitensky N. Usefulness of the Berg Balance Scale in stroke rehabilitation: a systematic review. Phys Ther. 2008;88(5):559–66. Epub 2008/02/23. doi: 10.2522/ptj.20070205 . 18292215

[13] Jaspers E, Desloovere K, Bruyninckx H, Klingels K, Molenaers G, Aertbelien E, et al. Three-dimensional upper limb movement characteristics in children with hemiplegic cerebral palsy and typically developing children. Res Dev Disabil. 2011;32(6):2283–94. Epub 2011/08/25. doi: 10.1016/j.ridd.2011.07.038 . 21862283

[14] Blenkinsop GM, Pain MTG, Hiley MJ. Balance control strategies during perturbed and unperturbed balance in standing and handstand. R Soc Open Sci. 2017;4(7):161018. Epub 2017/08/10. doi: 10.1098/rsos.161018 ; PubMed Central PMCID: PMC5541526. 28791131 PMC5541526

[15] Galna B, Barry G, Jackson D, Mhiripiri D, Olivier P, Rochester L. Accuracy of the Microsoft Kinect sensor for measuring movement in people with Parkinson’s disease. Gait Posture. 2014;39(4):1062–8. Epub 2014/02/25. doi: 10.1016/j.gaitpost.2014.01.008 . 24560691

[16] Dutta T. Evaluation of the Kinect sensor for 3-D kinematic measurement in the workplace. Appl Ergon. 2012;43(4):645–9. Epub 2011/10/25. doi: 10.1016/j.apergo.2011.09.011 . 22018839

[17] Mündermann L, Corazza S, Andriacchi TP. The evolution of methods for the capture of human movement leading to markerless motion capture for biomechanical applications. J Neuroeng Rehabil. 2006;3:6-. doi: 10.1186/1743-0003-3-6 . 16539701 PMC1513229

[18] Metcalf CD, Robinson R, Malpass AJ, Bogle TP, Dell TA, Harris C, et al. Markerless Motion Capture and Measurement of Hand Kinematics: Validation and Application to Home-Based Upper Limb Rehabilitation. IEEE Transactions on Biomedical Engineering. 2013;60(8):2184–92. doi: 10.1109/TBME.2013.2250286 23475333

[19] Khoshelham K, Elberink SO. Accuracy and resolution of Kinect depth data for indoor mapping applications. Sensors (Basel). 2012;12(2):1437–54. Epub 2012/02/01. doi: 10.3390/s120201437 . 22438718 PMC3304120

[20] Bleiweiss A, Kutliroff E. Markerless motion capture using a single depth sensor. 2009. doi: 10.1145/1667146.1667172

[21] Clark RA, Pua Y-H, Fortin K, Ritchie C, Webster KE, Denehy L, et al. Validity of the Microsoft Kinect for assessment of postural control. Gait & Posture. 2012;36(3):372–7. doi: 10.1016/j.gaitpost.2012.03.033 22633015

[22] Shotton J, Fitzgibbon A, Cook M, Sharp T, Finocchio M, Moore R, et al. Real-Time Human Pose Recognition in Parts from Single Depth Images. In: Cipolla R, Battiato S, Farinella GM, editors. Machine Learning for Computer Vision. Berlin, Heidelberg: Springer Berlin Heidelberg; 2013. p. 119–35.

[23] Pfister A, West AM, Bronner S, Noah JA. Comparative abilities of Microsoft Kinect and Vicon 3D motion capture for gait analysis. J Med Eng Technol. 2014;38(5):274–80. Epub 2014/06/01. doi: 10.3109/03091902.2014.909540 . 24878252

[24] Mentiplay BF, Perraton LG, Bower KJ, Pua YH, McGaw R, Heywood S, et al. Gait assessment using the Microsoft Xbox One Kinect: Concurrent validity and inter-day reliability of spatiotemporal and kinematic variables. J Biomech. 2015;48(10):2166–70. Epub 2015/06/13. doi: 10.1016/j.jbiomech.2015.05.021 . 26065332

[25] Müller B, Ilg W, Giese MA, Ludolph N. Validation of enhanced kinect sensor based motion capturing for gait assessment. PLOS ONE. 2017;12(4):e0175813. doi: 10.1371/journal.pone.0175813 28410413 PMC5391956

[26] Oh J, Kuenze C, Jacopetti M, Signorile JF, Eltoukhy M. Validity of the Microsoft Kinect() in assessing spatiotemporal and lower extremity kinematics during stair ascent and descent in healthy young individuals. Med Eng Phys. 2018;60:70–6. Epub 2018/08/12. doi: 10.1016/j.medengphy.2018.07.011 . 30097314

[27] Pehlivan N, Tunca C, Salur G, Ersoy C, editors. Gait analysis using kinect: Towards in-home gait analysis. 2017 25th Signal Processing and Communications Applications Conference (SIU); 2017 15–18 May 2017.

[28] Bowen DJ, Kreuter M, Spring B, Cofta-Woerpel L, Linnan L, Weiner D, et al. How we design feasibility studies. Am J Prev Med. 2009;36(5):452–7. doi: 10.1016/j.amepre.2009.02.002 . 19362699 PMC2859314

[29] Bannigan K, Watson R. Reliability and validity in a nutshell. Journal of Clinical Nursing. 2009;18(23):3237–43. doi: 10.1111/j.1365-2702.2009.02939.x 19930083

[30] Strauss ME, Smith GT. Construct validity: advances in theory and methodology. Annu Rev Clin Psychol. 2009;5:1–25. doi: 10.1146/annurev.clinpsy.032408.153639 . 19086835 PMC2739261

[31] The SAGE Encyclopedia of Educational Research, Measurement, and Evaluation. 2018. doi: 10.4135/9781506326139

[32] Furtado S, Godfrey A, Del Din S, Rochester L, Gerrand C. Free-living monitoring of ambulatory activity after treatments for lower extremity musculoskeletal cancers using an accelerometer-based wearable—a new paradigm to outcome assessment in musculoskeletal oncology? Disabil Rehabil. 2022:1–10. Epub 2022/06/17. doi: 10.1080/09638288.2022.2083701 . 35710327

[33] In J. Introduction of a pilot study. Korean J Anesthesiol. 2017;70(6):601–5. Epub 20171114. doi: 10.4097/kjae.2017.70.6.601 ; PubMed Central PMCID: PMC5716817. 29225742 PMC5716817

[34] Raptis M, Kirovski D, Hoppe H. Real-time classification of dance gestures from skeleton animation. Vancouver, British Columbia, Canada: Association for Computing Machinery; 2011. 147–56 p.

[35] Ferrell BR, Dow KH, Grant M. Measurement of the quality of life in cancer survivors. Qual Life Res. 1995;4(6):523–31. Epub 1995/12/01. doi: 10.1007/BF00634747 . 8556012

[36] Bornbaum CC, Doyle PC, Skarakis-Doyle E, Theurer JA. A critical exploration of the International Classification of Functioning, Disability, and Health (ICF) framework from the perspective of oncology: recommendations for revision. Journal of multidisciplinary healthcare. 2013;6:75–86. Epub 2013/03/26. doi: 10.2147/JMDH.S40020 ; PubMed Central PMCID: PMC3596126. 23526147 PMC3596126

[37] McDougall J, Wright V, Rosenbaum P. The ICF model of functioning and disability: incorporating quality of life and human development. Developmental neurorehabilitation. 2010;13(3):204–11. Epub 2010/05/11. doi: 10.3109/17518421003620525 . 20450470

[38] Hurvitz EA, Richardson JK, Werner RA, Ruhl AM, Dixon MR. Unipedal stance testing as an indicator of fall risk among older outpatients. Archives of Physical Medicine and Rehabilitation. 2000;81(5):587–91. doi: 10.1016/s0003-9993(00)90039-x 10807096

[39] White L, Hartnell N, Hennessy M, Mullan J. The Impact of an Intact Infrapatellar Fat Pad on Outcomes after Total Knee Arthroplasty. Advances in Orthopedic Surgery. 2015;2015:817906. doi: 10.1155/2015/817906

[40] Frykberg GE, Häger CK. Movement analysis of sit-to-stand–research informing clinical practice. Physical Therapy Reviews. 2015;20(3):156–67. doi: 10.1179/1743288X15Y.0000000005

[41] Ku PX, Abu Osman NA, Wan Abas WA. Balance control in lower extremity amputees during quiet standing: a systematic review. Gait Posture. 2014;39(2):672–82. Epub 2013/12/18. doi: 10.1016/j.gaitpost.2013.07.006 . 24331296

[42] Aksnes LH, Bauer HC, Jebsen NL, Folleras G, Allert C, Haugen GS, et al. Limb-sparing surgery preserves more function than amputation: a Scandinavian sarcoma group study of 118 patients. The Journal of bone and joint surgery British volume. 2008;90(6):786–94. Epub 2008/06/10. doi: 10.1302/0301-620X.90B6.19805 . 18539673

[43] Davis AM, Sennik S, Griffin AM, Wunder JS, O’Sullivan B, Catton CN, et al. Predictors of functional outcomes following limb salvage surgery for lower-extremity soft tissue sarcoma. Journal of surgical oncology. 2000;73(4):206–11. Epub 2000/05/08. doi: 10.1002/(sici)1096-9098(200004)73:4&lt;206::aid-jso4&gt;3.0.co;2-5 . 10797333

[44] Rantz M, Skubic M, Abbott C, Galambos C, Popescu M, Keller J, et al. Automated In-Home Fall Risk Assessment and Detection Sensor System for Elders. Gerontologist. 2015;55 Suppl 1(Suppl 1):S78–S87. doi: 10.1093/geront/gnv044 . 26055784 PMC4566912

[45] Kapoor A, Guha S, Kanti Das M, Goswami KC, Yadav R. Digital healthcare: The only solution for better healthcare during COVID-19 pandemic? Indian Heart Journal. 2020. doi: 10.1016/j.ihj.2020.04.001 32534691 PMC7151273

