# Supplementary material for: Feasibility of using low-cost markerless motion capture for assessing functional outcomes after lower extremity musculoskeletal cancer surgery
Source: PLoS One. 2024 Mar 28;19(3):e0300351. doi: 10.1371/journal.pone.0300351 (PMC10977781; doi:10.1371/journal.pone.0300351)
Supplement: S1 Checklist — (DOCX) [file pone.0300351.s001.docx]

STROBE Statement—checklist of items that should be included in reports of observational studies

|  | Item No. | Recommendation | Page  No. | Relevant text from manuscript |
| --- | --- | --- | --- | --- |
| **Title and abstract** | 1 | (*a*) Indicate the study’s design with a commonly used term in the title or the abstract | 3 | Under Methods section: Cross-sectional study |
|  |  | (*b*) Provide in the abstract an informative and balanced summary of what was done and what was found | 3 and 4 | Under Methods and Results sections |
| Introduction | | | |  |
| Background/rationale | 2 | Explain the scientific background and rationale for the investigation being reported | 5 and 6 | Under second and third paragraphs of introduction |
| Objectives | 3 | State specific objectives, including any prespecified hypotheses | 6 | Line 122 |
| Methods | | | |  |
| Study design | 4 | Present key elements of study design early in the paper | 7 | Under section 2.2.  Study Design: Cross-sectional Pilot and Feasibility study |
| Setting | 5 | Describe the setting, locations, and relevant dates, including periods of recruitment, exposure, follow-up, and data collection | 7-11 | Under section 2.3, 2.4, 2.5 and 2.7  Under Methods section, Equipment, test performed and assessment using video, scales |
| Participants | 6 | (*a*) *Cohort study*—Give the eligibility criteria, and the sources and methods of selection of participants. Describe methods of follow-up  *Case-control study*—Give the eligibility criteria, and the sources and methods of case ascertainment and control selection. Give the rationale for the choice of cases and controls  *Cross-sectional study*—Give the eligibility criteria, and the sources and methods of selection of participants | 7  Cross-sectional study | Under section 2.1, Additional details about the approach to recruitment and eligibility criteria can be found in a previous publication (10). Readers have been directed to this publication in the manuscript text. |
|  |  | (*b*) *Cohort study*—For matched studies, give matching criteria and number of exposed and unexposed  *Case-control study*—For matched studies, give matching criteria and the number of controls per case | N/A | N/A |
| Variables | 7 | Clearly define all outcomes, exposures, predictors, potential confounders, and effect modifiers. Give diagnostic criteria, if applicable | 8 | Under sections 2.2 |
| Data sources/ measurement | 8* | For each variable of interest, give sources of data and details of methods of assessment (measurement). Describe comparability of assessment methods if there is more than one group | 10-13 | Under table 1, and sections 2.7-2.10 |
| Bias | 9 | Describe any efforts to address potential sources of bias | 7-13 | Under sections 2.2, and 2.6-2.10 |
| Study size | 10 | Explain how the study size was arrived at | 7 | Under section 2.1 and additional details about the approach to recruitment, sample size and eligibility criteria can be found in a previous publication (10). Readers have been directed to this publication in the manuscript text. |

Continued on next page

| Quantitative variables | 11 | Explain how quantitative variables were handled in the analyses. If applicable, describe which groupings were chosen and why | 10-13 | Under sections 2.3 – 2.11 and in Table 1 |
| --- | --- | --- | --- | --- |
| Statistical methods | 12 | (*a*) Describe all statistical methods, including those used to control for confounding | 13 | Under section 2.11 |
|  |  | (*b*) Describe any methods used to examine subgroups and interactions | 8-9 | Under section 2.6 – 2.11 |
|  |  | (*c*) Explain how missing data were addressed | --- | --- |
|  |  | (*d*) *Cohort study*—If applicable, explain how loss to follow-up was addressed  *Case-control study*—If applicable, explain how matching of cases and controls was addressed  *Cross-sectional study*—If applicable, describe analytical methods taking account of sampling strategy | 7 | Under section 2.11 |
|  |  | (*e*) Describe any sensitivity analyses | --- | --- |
| Results | | | | |
| Participants | 13* | (a) Report numbers of individuals at each stage of study—eg numbers potentially eligible, examined for eligibility, confirmed eligible, included in the study, completing follow-up, and analysed | 14 | Under section 3.1 |
|  |  | (b) Give reasons for non-participation at each stage | 14 | Under section 3.1 |
|  |  | (c) Consider use of a flow diagram | 14 | Under section 3.1. Flowchart is presented in a recent publication by our group (10) and readers have been directed to this publication in the manuscript text. |
| Descriptive data | 14* | (a) Give characteristics of study participants (eg demographic, clinical, social) and information on exposures and potential confounders | 14 | Under section 3.1 and in Table 2. Further details are also present in the recent publication (10) and readers have been directed to this publication in the manuscript text. |
|  |  | (b) Indicate number of participants with missing data for each variable of interest | -- | ---- |
|  |  | (c) *Cohort study*—Summarise follow-up time (eg, average and total amount) | N/A | N/A |
| Outcome data | 15* | *Cohort study*—Report numbers of outcome events or summary measures over time | N/A | N/A |
|  |  | *Case-control study—*Report numbers in each exposure category, or summary measures of exposure | N/A | N/A |
|  |  | *Cross-sectional study—*Report numbers of outcome events or summary measures | 15-17 | Under section 3.3 and Table 2 |
| Main results | 16 | (*a*) Give unadjusted estimates and, if applicable, confounder-adjusted estimates and their precision (eg, 95% confidence interval). Make clear which confounders were adjusted for and why they were included | 14-21 | Under sections 3.3 a, b and c and in Tables 2, 3 and 4 |
|  |  | (*b*) Report category boundaries when continuous variables were categorized | 15-17 | In table 2 |
|  |  | (*c*) If relevant, consider translating estimates of relative risk into absolute risk for a meaningful time period | ---- | --- |

Continued on next page

| Other analyses | 17 | Report other analyses done—eg analyses of subgroups and interactions, and sensitivity analyses | 14-22 | Under section 3.3, a, b and c. and In Table 2, 3 and 4 |
| --- | --- | --- | --- | --- |
| Discussion | | | | |
| Key results | 18 | Summarise key results with reference to study objectives | 23 | Under section 4.1 |
| Limitations | 19 | Discuss limitations of the study, taking into account sources of potential bias or imprecision. Discuss both direction and magnitude of any potential bias | 26 | Under section 4.2, sub-heading *Agreement of MMC with Manual Techniques* |
| Interpretation | 20 | Give a cautious overall interpretation of results considering objectives, limitations, multiplicity of analyses, results from similar studies, and other relevant evidence | 29-31 | Under section 4.4 |
| Generalisability | 21 | Discuss the generalisability (external validity) of the study results | 30 | Last paragraph of discussion in section 4.4 |
| Other information | |  | | |
| Funding | 22 | Give the source of funding and the role of the funders for the present study and, if applicable, for the original study on which the present article is based | 33 | Under Section of Funding in the main manuscript and under funding information in the full submission (current funding resources list) |

*Give information separately for cases and controls in case-control studies and, if applicable, for exposed and unexposed groups in cohort and cross-sectional studies.

**Note:** An Explanation and Elaboration article discusses each checklist item and gives methodological background and published examples of transparent reporting. The STROBE checklist is best used in conjunction with this article (freely available on the Web sites of PLoS Medicine at http://www.plosmedicine.org/, Annals of Internal Medicine at http://www.annals.org/, and Epidemiology at http://www.epidem.com/). Information on the STROBE Initiative is available at www.strobe-statement.org.
