# Supplementary figures and images for: Feasibility of using low-cost markerless motion capture for assessing functional outcomes after lower extremity musculoskeletal cancer surgery
Source: PLoS One. 2024 Mar 28;19(3):e0300351. doi: 10.1371/journal.pone.0300351 (PMC10977781; doi:10.1371/journal.pone.0300351)

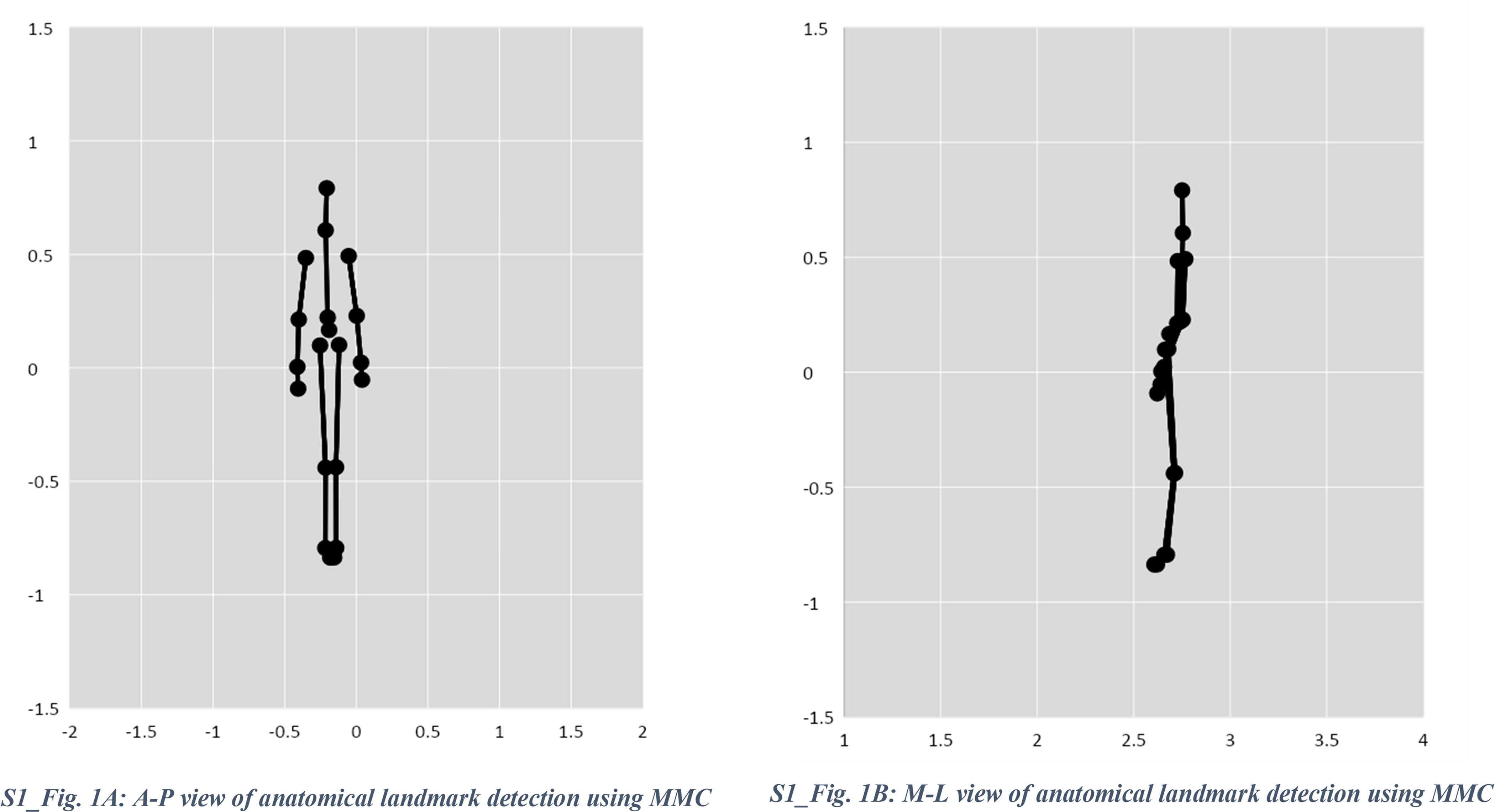

Supplement: S1 Fig — A: A-P view of anatomical landmark detection using MMC. B: M-L view of anatomical landmark detection using MMC. (TIF) [file pone.0300351.s002.tif]

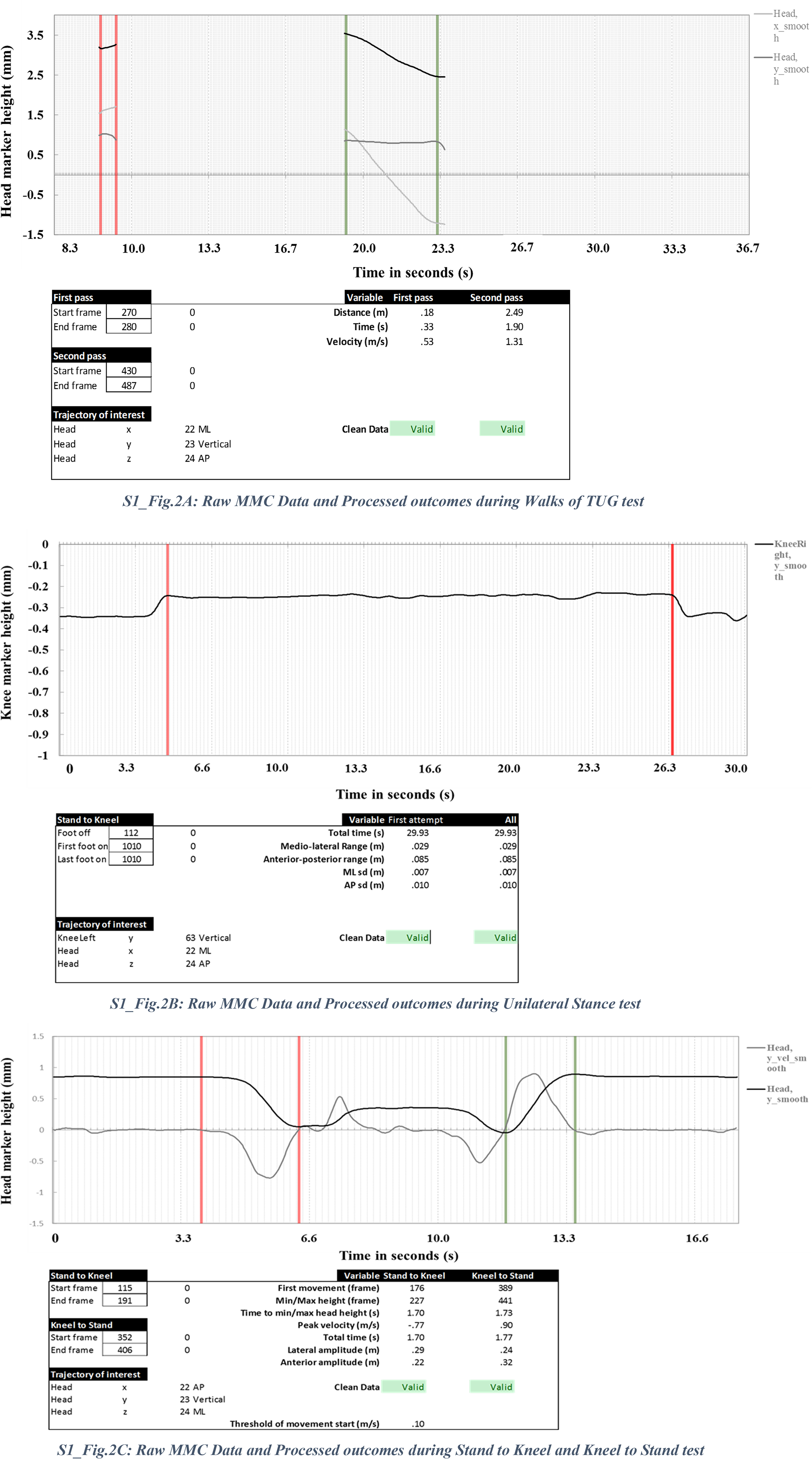

Supplement: S2 Fig — A: Raw MMC Data and Processed outcomes during walks of TUG test. B: Raw MMC Data and Processed outcomes during Unilateral Stance test. C: Raw MMC Data and Processed outcomes during Stand to Kneel and Kneel to Stand test. (TIF) [file pone.0300351.s003.tif]
