# Supplementary material for: Feasibility of using low-cost markerless motion capture for assessing functional outcomes after lower extremity musculoskeletal cancer surgery
Source: PLoS One. 2024 Mar 28;19(3):e0300351. doi: 10.1371/journal.pone.0300351 (PMC10977781; doi:10.1371/journal.pone.0300351)
Supplement: S1 Table — (PDF) [file pone.0300351.s004.pdf]

S1_Table1_pdf : Skeletal Tracking Data

Activity Assessed	Case Study Analysis of Skeletal Tracking Data
**1) Unilateral Stance test**	Patient A was a 77-year-old male diagnosed with a metastatic bone tumour in his pelvis and had undergone a left proximal femur resection with insertion of a modular Stanmore bipolar hemi-arthroplasty implant. A visual analysis of the skeletal tracker data of Left sided unilateral stance was performed for Patient A. It was found that the patient while clearing the right foot off the floor and transferring weight onto their left lower limb used postural compensations, such as moving hands in space and leaning towards the left side using their trunk in an attempt to maintain balance. After a struggle, the patient finally placed back their right foot on the floor. The patient stood on the left leg for a short period of time as captured by the Kinect skeletal tracker. 2.90 s was the recorded time. (Skeletal Tracking Video of Patient A).
**2) STK and TKS tests**	Patient B was a 43-year-old male diagnosed with a myxofibrosarcoma (soft tissue sarcoma) in the right lower leg who underwent a wide excision and free antero-lateral thigh flap reconstruction. A visual analysis of the skeletal tracker data of kneeling of Patient B demonstrates the movements of the patient during STK and KTS. The patient managed to perform STK more easily, but while rising from KTS faced some struggle. The patient went slightly off balance while performing KTS as moved around the place and placed a foot behind when returning to the standing position. (Skeletal Tracking Video of Patient B).
