# Supplementary material for: Feasibility of using low-cost markerless motion capture for assessing functional outcomes after lower extremity musculoskeletal cancer surgery
Source: PLoS One. 2024 Mar 28;19(3):e0300351. doi: 10.1371/journal.pone.0300351 (PMC10977781; doi:10.1371/journal.pone.0300351)
Supplement: Attachment — Submitted filename: Response to Reviewers.docx [file pone.0300351.s005.docx]

**View Letter**

| **To:** | "Sherron Furtado" s.furtado@ucl.ac.uk |
| --- | --- |
| **From:** | "PLOS ONE" plosone@plos.org |
| **Subject:** | PLOS ONE Decision: Revision required [PONE-D-23-14657] |

PONE-D-23-14657

Feasibility of Using Low-cost Markerless Motion Capture for Assessing Functional Outcomes after Lower Extremity Musculoskeletal Cancer Surgery

PLOS ONE

Dear Dr. Furtado,

Thank you for submitting your manuscript to PLOS ONE. After careful consideration, we feel that it has merit but does not fully meet PLOS ONE’s publication criteria as it currently stands. Therefore, we invite you to submit a revised version of the manuscript that addresses the points raised during the review process.

Please submit your revised manuscript by Sep 08 2023 11:59PM. If you will need more time than this to complete your revisions, please reply to this message or contact the journal office at [plosone@plos.org](mailto:plosone@plos.org). When you're ready to submit your revision, log on to https://www.editorialmanager.com/pone/ and select the 'Submissions Needing Revision' folder to locate your manuscript file.

Please include the following items when submitting your revised manuscript:

- A rebuttal letter that responds to each point raised by the academic editor and reviewer(s). You should upload this letter as a separate file labeled 'Response to Reviewers'.
- A marked-up copy of your manuscript that highlights changes made to the original version. You should upload this as a separate file labeled 'Revised Manuscript with Track Changes'.
- An unmarked version of your revised paper without tracked changes. You should upload this as a separate file labeled 'Manuscript'.

If you would like to make changes to your financial disclosure, please include your updated statement in your cover letter. Guidelines for resubmitting your figure files are available below the reviewer comments at the end of this letter.

If applicable, we recommend that you deposit your laboratory protocols in protocols.io to enhance the reproducibility of your results. Protocols.io assigns your protocol its own identifier (DOI) so that it can be cited independently in the future. For instructions see: <https://journals.plos.org/plosone/s/submission-guidelines#loc-laboratory-protocols>. Additionally, PLOS ONE offers an option for publishing peer-reviewed Lab Protocol articles, which describe protocols hosted on protocols.io. Read more information on sharing protocols at <https://plos.org/protocols?utm_medium=editorial-email&utm_source=authorletters&utm_campaign=protocols>.

We look forward to receiving your revised manuscript.

Kind regards,

Roberto Di Marco, PhD
Academic Editor
PLOS ONE

Journal Requirements:

When submitting your revision, we need you to address these additional requirements.

1. Please ensure that your manuscript meets PLOS ONE's style requirements, including those for file naming. The PLOS ONE style templates can be found at

https://journals.plos.org/plosone/s/file?id=wjVg/PLOSOne_formatting_sample_main_body.pdf and 
<https://journals.plos.org/plosone/s/file?id=ba62/PLOSOne_formatting_sample_title_authors_affiliations.pdf>

A1. Thank you for your comment, we have now addressed this. We have edited all figures and tables, and how they appear in the text. We have added headings and captions and saved the file as per journal requirements. We have also addressed the author/title page with requirements mentioned in links above.

2. We note that the grant information you provided in the ‘Funding Information’ and ‘Financial Disclosure’ sections do not match.

When you resubmit, please ensure that you provide the correct grant numbers for the awards you received for your study in the ‘Funding Information’ section.

A2. Thanks for pointing this out. We have edited the information in the manuscript text to address this and have added the following statements on Page 31 of the main manuscript, under the section of Funding information.

**Funding Information**

“The institution of one or more of the authors (CG, SF) has received, during the study period, funding from the Children with Cancer UK Charity (Grant number: 2012/139) https://www.childrenwithcancer.org.uk/childhood-cancer-info/we-fundresearch/projects-we-fund/assessing-physical-function/, Sarcoma UK Charity (Grant number: 007.2012), and Shear’s Foundation and Research and Capability (RCF).”

3. Thank you for stating the following in the Competing Interests section:

"The institution of one or more of the authors (AG, LR) has received, during the study period, funding from the National Institute for Health Research (NIHR) Newcastle Biomedical Research Centre and Unit based at Newcastle upon Tyne Hospitals NHS Foundation Trust and
Newcastle University. The institution of one or more of the authors (AG, SDD, LR) has received, during the study period, funding from the NIHR Newcastle Clinical Research Faculty infrastructure funding. The institution of one or more of the authors (CG, SF) has received, during the study period, funding from the Children with Cancer UK Charity, Sarcoma UK Charity, and Shear’s Foundation and Research and Capability (RCF).

All ICMJE Conflict of Interest Forms for authors are on file with the publication and can be viewed on request.

Each author certifies that his or her institution approved the human protocol for this investigation and that all investigations were conducted in conformity with ethical principles of research.

This work was performed at the major clinic sites and Human Movement Room at the North of England Bone and Soft Tissue Tumor Service, Newcastle Upon Tyne
Hospitals NHS Foundation Trust, UK."

Please confirm that this does not alter your adherence to all PLOS ONE policies on sharing data and materials, by including the following statement: ""This does not alter our adherence to  PLOS ONE policies on sharing data and materials.” (as detailed online in our guide for authors http://journals.plos.org/plosone/s/competing-interests).  If there are restrictions on sharing of data and/or materials, please state these.

Please note that we cannot proceed with consideration of your article until this information has been declared.

Please include your updated Competing Interests statement in your cover letter; we will change the online submission form on your behalf.

A3. Thank you for pointing this out. To address this, we have added the following sentence in the covering letter and manuscript title page:

“As this study involves participation by human research participants, the data sharing is therefore bound by the National Research Ethics Committee (NREC) approval for the study which we need to abide by.”

4. We noted in your submission details that a portion of your manuscript may have been presented or published elsewhere:

"This Paper relates to two published studies on accelerometery as analyses different datasets from the same study sample, but is different from those published as this paper investigates a different technology and approach MMC. I have included the two related papers in this submission."

Please clarify whether this publication was peer-reviewed and formally published. If this work was previously peer-reviewed and published, in the cover letter please provide the reason that this work does not constitute dual publication and should be included in the current manuscript.

A4. Thank you for your comment. Yes, this publication was peer-reviewed and formally published. We have provided the reason in the covering letter as requested. We have edited the following statement in the covering letter.

“This Paper relates to two published studies on accelerometery as it analyses different datasets from the same study sample. However, it is different from those published as this paper investigates a different technology and approach (ie MMC). The other publications do not include data about MMC which is the focus of the current distinct manuscript.”

5. In your Data Availability statement, you have not specified where the minimal data set underlying the results described in your manuscript can be found. PLOS defines a study's minimal data set as the underlying data used to reach the conclusions drawn in the manuscript and any additional data required to replicate the reported study findings in their entirety. All PLOS journals require that the minimal data set be made fully available. For more information about our data policy, please see http://journals.plos.org/plosone/s/data-availability.

Upon re-submitting your revised manuscript, please upload your study’s minimal underlying data set as either Supporting Information files or to a stable, public repository and include the relevant URLs, DOIs, or accession numbers within your revised cover letter. For a list of acceptable repositories, please see http://journals.plos.org/plosone/s/data-availability#loc-recommended-repositories. Any potentially identifying patient information must be fully anonymized.

Important: If there are ethical or legal restrictions to sharing your data publicly, please explain these restrictions in detail. Please see our guidelines for more information on what we consider unacceptable restrictions to publicly sharing data: http://journals.plos.org/plosone/s/data-availability#loc-unacceptable-data-access-restrictions. Note that it is not acceptable for the authors to be the sole named individuals responsible for ensuring data access.

We will update your Data Availability statement to reflect the information you provide in your cover letter.

A5. This study involves human research participant data and contains sensitive data. National Research Ethics Committee (NREC) approval was required to run the study, which restricts sharing the data publicly. We are bound by the following ethics proposal:

“Personal data relating to patients will be held securely within the research team and in accordance with Data Protection legislation. All trust research staff are trained and comply with the NUTH Information Governance policy. Personal data by trust research staff will be handled in accordance with the NHS Code of Confidentiality and under the requirements of ICH GCP. Following completion of the study data will be archived in accordance with Trust Archival Procedures.

All patients will be given a study code and patient identifiers will be removed from the data to be analysed. Hard copies of the data would be kept in a locked office and all databases will be password protected on NHS Trust computers which are kept in a locked office. Data will be anonymised before passing to Newcastle University researchers.

As this study was conducted in the NHS, the ethics for this study restricts the access to patient data. The patient data is restricted to research staff listed on the Site Delegation Log to perform research activities as defined and authorized by the Principal Investigator. Only designated health care staff can have access to medical records, if necessary to verify clinical details. We will consult with our ethics committee and sponsor, and if allowed, we may be able to make the anonymised data sets available upon request."

6. We note that Figure S1 Fig 1A in your submission contain copyrighted images. All PLOS content is published under the Creative Commons Attribution License (CC BY 4.0), which means that the manuscript, images, and Supporting Information files will be freely available online, and any third party is permitted to access, download, copy, distribute, and use these materials in any way, even commercially, with proper attribution. For more information, see our copyright guidelines: http://journals.plos.org/plosone/s/licenses-and-copyright.

We require you to either (1) present written permission from the copyright holder to publish these figures specifically under the CC BY 4.0 license, or (2) remove the figures from your submission:

(1) You may seek permission from the original copyright holder of Figure S1 Fig 1A to publish the content specifically under the CC BY 4.0 license.

We recommend that you contact the original copyright holder with the Content Permission Form (http://journals.plos.org/plosone/s/file?id=7c09/content-permission-form.pdf) and the following text:
“I request permission for the open-access journal PLOS ONE to publish XXX under the Creative Commons Attribution License (CCAL) CC BY 4.0 (http://creativecommons.org/licenses/by/4.0/). Please be aware that this license allows unrestricted use and distribution, even commercially, by third parties. Please reply and provide explicit written permission to publish XXX under a CC BY license and complete the attached form.”

Please upload the completed Content Permission Form or other proof of granted permissions as an ""Other"" file with your submission.

In the figure caption of the copyrighted figure, please include the following text: “Reprinted from [ref] under a CC BY license, with permission from [name of publisher], original copyright [original copyright year].”

(2) If you are unable to obtain permission from the original copyright holder to publish these figures under the CC BY 4.0 license or if the copyright holder’s requirements are incompatible with the CC BY 4.0 license, please either i) remove the figure or ii) supply a replacement figure that complies with the CC BY 4.0 license. Please check copyright information on all replacement figures and update the figure caption with source information.

If applicable, please specify in the figure caption text when a figure is similar but not identical to the original image and is therefore for illustrative purposes only.

A6. We are unable to obtain permission from the original copyright holder to publish these figures, so we have removed Figure S1 Fig 1A from our submission. The manuscript has been edited and figures renumbered to reflect this.

**Additional Editor Comments:**

Specifically, Reviewers are asking more clarity and details at some stages of the manuscript. The manuscript and the usability of the propose system would also benefit from a clear definition of the equipment performances (accuracy and precision of MMC system -as taken from literature would be acceptable-, influence of depth sensor on keypoints tracking, robustness to presence of third subjects, ...).

[Note: HTML markup is below. Please do not edit.]

Reviewers' comments:

We sincerely thank PLOS, the editor and the reviewers for taking time to review this manuscript. We believe we have addressed points above by responding to all comments of reviewers in the sections below. We have labelled the line numbers and page numbers based on Simple Markup selected in the Review tab in the tracked changes document. This will also apply to the clean document. Many thanks to all the reviewers once again for providing these invaluable comments, as these have improved the manuscript.

Reviewer's Responses to Questions

**Comments to the Author**

1. Is the manuscript technically sound, and do the data support the conclusions?

The manuscript must describe a technically sound piece of scientific research with data that supports the conclusions. Experiments must have been conducted rigorously, with appropriate controls, replication, and sample sizes. The conclusions must be drawn appropriately based on the data presented.

Reviewer #1: No

Reviewer #2: Partly

Reviewer #3: Yes

2. Has the statistical analysis been performed appropriately and rigorously?

Reviewer #1: I Don't Know

Reviewer #2: Yes

Reviewer #3: Yes

3. Have the authors made all data underlying the findings in their manuscript fully available?

The [PLOS Data policy](http://www.plosone.org/static/policies.action#sharing) requires authors to make all data underlying the findings described in their manuscript fully available without restriction, with rare exception (please refer to the Data Availability Statement in the manuscript PDF file). The data should be provided as part of the manuscript or its supporting information, or deposited to a public repository. For example, in addition to summary statistics, the data points behind means, medians and variance measures should be available. If there are restrictions on publicly sharing data—e.g. participant privacy or use of data from a third party—those must be specified.

Reviewer #1: Yes

Reviewer #2: Yes

Reviewer #3: Yes

4. Is the manuscript presented in an intelligible fashion and written in standard English?

PLOS ONE does not copyedit accepted manuscripts, so the language in submitted articles must be clear, correct, and unambiguous. Any typographical or grammatical errors should be corrected at revision, so please note any specific errors here.

Reviewer #1: No

Reviewer #2: Yes

Reviewer #3: Yes

5. Review Comments to the Author

Please use the space provided to explain your answers to the questions above. You may also include additional comments for the author, including concerns about dual publication, research ethics, or publication ethics. (Please upload your review as an attachment if it exceeds 20,000 characters)

Reviewer #1:

Q1. The authors used Markerless Motion Capture (MCC) to assess functional outcomes after sarcoma surgery in a cross section of 34 patients. The authors conclude that walking, single leg stance and kneeling assessment was feasible, acceptable and ‘clinically useful’.
This is an interesting technology that could have potential to improve our understanding of functional limitations after sarcoma surgery.
However, the manuscript fails to clearly communicate and interpret the study data. In addition, the study population is extremely heterogeneous (soft-tissue sarcoma, bone sarcoma, amputation, limb-salvage, all included). A more focused patient population would significantly improve the value of this study with respect to clinical relevance.

A1. Thank you for taking the time to review this manuscript and for your invaluable comments. We agree that this is an interesting technology, and apologise that the manuscript has not clearly communicated and interpreted the data. To address this, we have revisited the results and discussion sections in the manuscript, to make the clinical interpretation of the paper clearer. As you rightly pointed out, this is an important piece of work for people facing functional limitations after sarcoma surgery, as for the first time the feasibility of such an approach has been investigated for patients with a rare disease like sarcoma.

This study highlights that it is not only feasible to capture new functional outcomes in this patient group, but also that the measures extracted are comparable to the literature, can differentiate between patient sub-groups and agree disease-specific clinical scales and standard assessments in clinic. This is promising for validation in a future larger study. The functional outcomes captured have the potential to identify physical limitations individual to the patient, inform targeted rehabilitation interventions thereby ensuring a delivery of high-quality evidence-based care and better outcomes for patients with sarcomas.

We have added content on page 16, 20 and 21 in the results section to address your comment and clearly communicate the study data.

We would also like to bring to your attention, the discussion section on pages 26-30, which we have significantly revised to appropriately interpret the data and emphasise the clinical relevance of this work and how MMC can revolutionise the delivery of healthcare for this rare disease. Clinical implications in the discussion have been revised to highlight the changes MMC can make to clinical practice.

To strengthen the clinical interpretation, we have revised the discussion under the sections of comparison with literature and between tumour sub-groups, investigating the relationships between established disease-specific scales and traditional assessments. This is reflected on lines 404 – 471.

Abstract
Q2. ¬ Line 73: (19-89) is this an age range, interquartile range? Standard deviation?

A2. 19-89 is the minimum value - maximum value of the age range. This has been edited on lines 82.

Q3. ¬ Line 75: please clarify ‘clinically useful data’.

A3. Thank you for this important comment. I have clarified this in the manuscript by adding further details on lines 91-93.

Q4¬ What do the ‘*’s mean in the abstract?

A4. I have clarified this by adding [* = statistical significance] on lines 88-89.

Q5¬ Overall, the abstract fails to connect the results of the analyses with the conclusions. What is the connection between the results and ‘novel knowledge’, ‘overcoming limitations’ and ‘guiding clinical management and rehabilitation’?

A5. Thanks for this important comment. I have now added statements in the abstract, to clearly link all these headings together. See revision in abstract on lines 82-96.

Introduction
Q6. ¬ Line 123: Please clarify ‘inexpensive’. What costs specifically are reduced with the Microsoft Kinect?

A6. This has been clarified in on lines 139-140.

Q7. ¬ Overall, the rationale for the study is well explained and well supported by background

information.

A7. Thank you for your comment.

Methods
Q8¬ Lines 138-139: Please clarify why a new paper is required from the data accessed, given the 2 previous publications.

A8. Thank you for this important comment. We have clarified this further on lines 172 to 178.

Q9¬ The pilot sample was one of convenience. That would exclude any statistical analysis. Therefore, statements such as ‘measures sensitive enough to significantly differentiate between tumour groups’, need further rationale/clarification. Same comment for lines 185-186.

A9. Thank you for your comment, although this was a pilot sample of convenience and therefore has inherent limitations; the results of this study showed important results. For instance, certain measures were successful in identifying differences between two clinical groups which was promising. We completely agree, a larger study with a purposive sampling strategy is required to confirm these findings, however the current study provides valuable knowledge and a contribution to the literature by demonstrating feasibility, significance, clinical trends and highlighting important relationships. We have clarified this in the discussion section lines 495-503 by recognising the limitations and proposing future work in this field. We have also avoided the use of words like “sensitive” in the manuscript, and in relevant sections of the manuscript including methods, results and discussion we have emphasized that this is pilot and feasibility work, which also explores early indicators of validity.

Q10¬ Lines 165-166: Please provide more information on the patient feedback survey.

A10. We have added additional sentences on Lines 212-216 to address your comment.

Q11¬ Lines 220-223: Statistical Analysis. Please clarify study power if stats were performed, with a sample size that was one of convenience.

A11. As this was a pilot and feasibility study, sample size and power calculations were not undertaken. We have included justification here and in the main text about why this feasibility and pilot study sample is important and how convenience sampling is an appropriate approach.

The purpose of the pilot study was to test the appropriateness and feasibility of the in-depth protocol of the MMC approach for the first time in a musculoskeletal tumour population. A convenience sample of 40 subjects (34 adults and 6 children) from the target population was used in the pilot study.

The sample size of 40 participants is a pragmatic choice although it is large enough (n > 30) to rely on the central limit theory. As the pilot study did not involve validation of MMC, 34 adult participants were thought sufficient to demonstrate variation in the target population.

As this study uses a new approach (MMC) in a cancer population, we needed to first test its feasibility, demonstrate early validity and explore known-group validity in our target population. In our descriptive analysis, we found sensible difference in patient sub-groups and sensible associations with known outcome measures,  which is promising. These results are important for a next larger study and will inform its design. The number of patients recruited was sufficient we felt to demonstrate the range of results in this varied population and generate hypotheses for testing in a larger study.

We have added relevant explanation/statements in the Methods and Discussion sections to clarify these points. In methods section, lines 184-191 and in the discussion sections, lines 495-503 and 514-516 support our explanation and clarify this further.

Results
Q12¬ Line 240: Please provide some information on the cohort that declined to participate.

A12 – Thank you for this important comment. Patients who primarily declined participation were those who lived far away from the specialist centre where assessments were undertaken. As this is a rare cancer and the North of England Bone and Soft tissue tumour service treats patients from the North of England and North-East, several patients attending clinics lived at 2-3 hours or more from the hospital. These were mainly patients who refused participation. I have added the details in lines 295-297.

¬ Line 251: Only 19 of 34 patients completed the survey. Please comment on this relatively poor response rate.

Thanks for highlighting this, we have explained this point in the discussion on lines 490-494. as follows

“Despite two reminders sent to patients, the response rate for the feedback points was found to be only 50% (19/34). This can introduce bias about acceptability and user-friendliness of MMC and results must be treated with caution. In a larger study, we will approach this issue by completing feedback forms with patients in clinic or on phone, rather than by post to ensure a good response rate is achieved.”

¬ Overall, the Results section is difficult to follow, with extensive technical jargon. If the data is meant to improve outcomes for orthopaedic oncology patients, the language should be appropriate for orthopaedic oncologists who treat them, not kinematic and kinetic specialists.

Thank you for this comment. This was not our intention as we wanted to our paper to be targeted mainly for Rehabilitation professionals treating sarcoma patients to return to their lives after major surgery. As you rightly pointed out, other multi-disciplinary team members treating these patients such as Orthopaedic Oncologists, Surgeons, and other clinicians dealing with such a complex group with an in-depth understanding of functional issues patients face, may also have an interest in the results.

We have edited this results section on pages 415-471 and 504-513 to make the message clearer for all health care professionals, to target all the audience providing treatment and follow-up effectively.

Discussion
¬ Lines 311-318: the study conclusions are strong, given that the data is not well interpreted for the reader. In addition, the low response rate weakens the ‘acceptability’ conclusions.

Thank you for this valuable comment. We have edited this to reflect the appropriate interpretation of this work in the opening discussion section on lines 404-413 and in the rest of the discussion and also in the conclusion section on page 533-547.

¬ Lines 401-425: This section, case studies, is the most useful for the clinician: how can this technology be used to improve patient outcomes?
¬ Overall, the Discussion is 3-4 X longer than appropriate. It is unfocused and disjointed. I suggest a complete rewrite.

Thanks for this important and very valuable comment. We have re-written this section to make it more focussed and shorter. Kindly see the discussion section from lines 403-532.

Conclusions
¬ The conclusions are difficult to assess, given the lack of clear interpretation of study results.

After re-writing the discission, we think the interpretation of study results is clearer. We have edited the conclusion to make this clearer too. The case studies are described in 509-513.

Reviewer #2: I congratulate authors on this new and promising tool that could help in monitoring effectivity of targeted physical therapy for sarcoma patients. These are my comments to authors :

Thanks for your positive comment.

1. The study was performed only on patients who had a lower limb surgery. Could the authors elaborate if any ongoing studies involve the evaluation of patients following surgery for upper limb sarcomas or not and do they think MMC could be equally effective in this group of patients.

A1. Thanks for this important point and agree, it would be of great value to test the MMC approach in upper limb sarcoma patients. There are currently no studies on MMC in Upper limb sarcoma patients.

2. Although sample size was justified based on the study being a pilot study (looking at feasibility, safety and patient compliance), statistical conclusions regarding convergent and discriminant validities based on this sample size may lack sufficient power.

A2. Thank you for this comment. We have explained that these are early indicators of convergent and divergent validity and completely agree that the study lacks sufficient power to draw definitive conclusions. However this pilot work will support a future larger study and will form a strong base to guiding the methodology and statistical approaches in the next steps. This has been explained in the discussion and we have edited the paper to reflect the main findings of feasibility of the approach and moved away from the discussion about validity. Kindly refer to sections 4.2 and 4.3 of the discussion from pages 26-29.

3. I suggest that in future continuing research, using healthy subjects as controls would be recommended.

A3. Thank you for this very valuable comment. We will enrol healthy subjects as controls in future continuing research and this has been references on line 291.

4. Seven patients were included in amputation group. Could you confirm if all 7 patients had the same type of amputation or not. An above knee amputation may have a functional outcome different from a below knee amputation.

A4. Thank you for your comment. The clinical details of the patient sample have been described in detail in a previous publication and referenced on line 291.

The levels of amputation were 1 hind-quarter amputation, 3 above-knee amputations and 3 below-knee amputations. We agree that an above knee would have different functional outcomes compared to a below knee amputation, but as this was a pilot study with very small numbers; requiring us to take the approach of clubbing patients into a single group. Agree with your comment, and therefore we have added additional sentences to explain this as a limitation in the study and have made a reference that future studies will enrol homogenous groups. This has been added on lines 495-503.

5. Although your study showed statistical significance in discriminating between Bone and soft tissue sarcomas as a matter of functional outcome, from clinical point of view , the difference in outcome would rely more on the extent of surgery and reconstruction rather than the primary diagnoses of the disease. The functional outcome may be the same in large sized sarcomas whether primarily affecting bone or soft tissues.

A5. Thank you for this invaluable comment. We have addressed this in the discussion section by adding an explanation on lines 439-445.

Reviewer #3: The manuscript presents an interesting markerless motion capture method to assess functional outcomes after sarcoma surgery. The work highlighted the feasibility of using markerless motion capture based on Kinect technology to assess impairments in patient movements. The study is relevant to be published in the journal.
However, in my opinion, I propose some suggestions that could improve this work.

It will be helpful to the reader if you could briefly describe the established sarcoma assessments TESS and MSTS (lines 91-92).

Thank you for your valuable comment. As requested, we have edited this information to briefly describe these established sarcoma assessments TESS and MSTS. The revisions have been made on lines 115-121.

I suggest to slightly elaborate the Introduction section (lines 117-121) to what concern the review of the current literature that use the Microsoft Kinect in movement analysis, adding also relevant recent works, e.g. from the last five years.

Thanks for this important comment. References from previous related work were already added, please refer to lines 136-149, but to address your comment we have also now added references to recent relevant work on lines 149-153.

The overall presentation of results is clear. The Authors also identify the limitations of their study. I suggest to modify at line 517-518 “current covid 19 pandemic” with “pandemic conditions” in order to keep it more general.

Thank you, we have addressed this. Please see lines 529-532.

Thanks once again for reviewing this manuscript.

6. PLOS authors have the option to publish the peer review history of their article ([what does this mean?](https://journals.plos.org/plosone/s/editorial-and-peer-review-process#loc-peer-review-history)). If published, this will include your full peer review and any attached files.


If you choose “no”, your identity will remain anonymous but your review may still be made public.

**Do you want your identity to be public for this peer review?** For information about this choice, including consent withdrawal, please see our [Privacy Policy](https://www.plos.org/privacy-policy).

Reviewer #1: No

Reviewer #2: **Yes:** ahmed mohamed el ghoneimy

Reviewer #3: No

[NOTE: If reviewer comments were submitted as an attachment file, they will be attached to this email and accessible via the submission site. Please log into your account, locate the manuscript record, and check for the action link "View Attachments". If this link does not appear, there are no attachment files.]

While revising your submission, please upload your figure files to the Preflight Analysis and Conversion Engine (PACE) digital diagnostic tool, <https://pacev2.apexcovantage.com/>. PACE helps ensure that figures meet PLOS requirements. To use PACE, you must first register as a user. Registration is free. Then, login and navigate to the UPLOAD tab, where you will find detailed instructions on how to use the tool. If you encounter any issues or have any questions when using PACE, please email PLOS at [figures@plos.org](mailto:figures@plos.org). Please note that Supporting Information files do not need this step.

*In compliance with data protection regulations, you may request that we remove your personal registration details at any time.* [*(Remove my information/details)*](https://www.editorialmanager.com/pone/login.asp?a=r)*. Please contact the publication office if you have any questions.*
